# A Model for the Fast Synchronous Oscillations of Firing Rate in Rat Suprachiasmatic Nucleus Neurons Cultured in a Multielectrode Array Dish

**DOI:** 10.1371/journal.pone.0106152

**Published:** 2014-09-05

**Authors:** Andrey R. Stepanyuk, Pavel V. Belan, Nikolai I. Kononenko

**Affiliations:** 1 Bogomoletz Institute of Physiology, Kiev, Ukraine; 2 State Key Laboratory of Molecular and Cellular Biology, Kiev, Ukraine; University of Alabama at Birmingham, United States of America

## Abstract

When dispersed and cultured in a multielectrode dish (MED), suprachiasmatic nucleus (SCN) neurons express fast oscillations of firing rate (FOFR; fast relative to the circadian cycle), with burst duration ∼10 min, and interburst interval varying from 20 to 60 min in different cells but remaining nevertheless rather regular in individual cells. In many cases, separate neurons in distant parts of the 1 mm recording area of a MED exhibited correlated FOFR. Neither the mechanism of FOFR nor the mechanism of their synchronization among neurons is known. Based on recent data implicating vasoactive intestinal polypeptide (VIP) as a key intercellular synchronizing agent, we built a model in which VIP acts as both a feedback regulator to generate FOFR in individual neurons, and a diffusible synchronizing agent to produce coherent electrical output of a neuronal network. In our model, VIP binding to its (VPAC_2_) receptors acts through G_s_ G-proteins to activate adenylyl cyclase (AC), increase intracellular cAMP, and open cyclic-nucleotide-gated (CNG) cation channels, thus depolarizing the cell and generating neuronal firing to release VIP. In parallel, slowly developing homologous desensitization and internalization of VPAC_2_ receptors terminates elevation of cAMP and thereby provides an interpulse silent interval. Through mathematical modeling, we show that this VIP/VPAC_2_/AC/cAMP/CNG-channel mechanism is sufficient for generating reliable FOFR in single neurons. When our model for FOFR is combined with a published model of synchronization of circadian rhythms based on VIP/VPAC_2_ and Per gene regulation synchronization of circadian rhythms is significantly accelerated. These results suggest that (a) auto/paracrine regulation by VIP/VPAC_2_ and intracellular AC/cAMP/CNG-channels are sufficient to provide robust FOFR and synchrony among neurons in a heterogeneous network, and (b) this system may also participate in synchronization of circadian rhythms.

## Introduction

Daily rhythms of sleep and wakefulness, physiology, and metabolism are coordinated by a brain clock located in the paired suprachiasmatic nuclei (SCN) [1]. The molecular machinery that drives circadian rhythmicity occurs in individual SCN neurons [2]–[4]. These neurons express self-sustained circadian oscillations driven by autoregulatory transcription–translation feedback loops [1], [5]. However, there is a wide range of evidence for functionally distinct cell populations within the SCN [6]–[9]. Some SCN cells are not endogenously rhythmic with respect to clock gene expression [9], and individual clock cells exhibit various phases and free-running periods [4]. Elucidating the mechanisms that synchronize a population of circadian oscillators displaying disparate periods is still a major unresolved issue in the field of circadian clock physiology. Various mechanisms, including gap junctions [10], synaptic contacts [11], and paracrine regulation [12] have been proposed as synchronizers, although these factors may act within different temporal and spatial frameworks, and the principles of them are largely unknown. Recent experimental evidence has shown that vasoactive intestinal polypeptide (VIP) is required for circadian synchrony in the SCN and behavior [13], and a corresponding mathematical model where VIP serves as a paracrine synchronizer for SCN neurons was developed [14]. When recording firing rates of dispersed rat SCN neurons in multi-electrode array dishes (MEDs), we discovered a new phenomenon: fast (relative to the circadian cycle) oscillations of firing rate (FOFR) with a duration of bursts ∼10 min and an interburst interval varying from 20 to 60 min in different cells but remaining rather regular in individual cells. We have hypothesized that the FOFR observed in cultured SCN neurons may contribute to synchronization of the circadian rhythm in the intact SCN [15]. Since mechanisms of FOFR generation remain unclear, we have used mathematical modeling to clarify these mechanisms. We have used the pulsatile activity in gonadotropin-releasing hormone (GnRH) neurons with a period about 30 min [16], [17] as a useful analogy in our modeling because: i) both SCN and GnRH neurons are located in the hypothalamus and one could expect similarity in their properties; ii) both single GnRH neurons and the network of GnRH neurons as a whole fire in a pulsatile manner with pulse duration ∼5 min and interpulse interval ∼30 min, closely matching the properties of FOFR in cultured SCN neurons; and iii) both circadian rhythmicity and synchrony in SCN neurons is partly mediated by vasoactive intestinal polypeptide (VIP) [13], a type of autocrine regulation of activity that is also found in GnRH neurons [12], [16], [17]. Here, we present a mathematical model to describe the FOFR in a single SCN cell and synchronization of a population of heterogeneous SCN neurons coupled through diffusible VIP in the extracellular medium. The goal of the present work is to show how a simple model can account for the FOFR in dispersed and cultured SCN neurons and for synchronization of their electrical activity. This work represents a step toward developing a multicellular, molecular model of the mammalian circadian clock exhibiting both fast oscillations of firing rate and circadian rhythms.

## Materials and Methods

### Basic assumptions of the model

**Figure 1:**
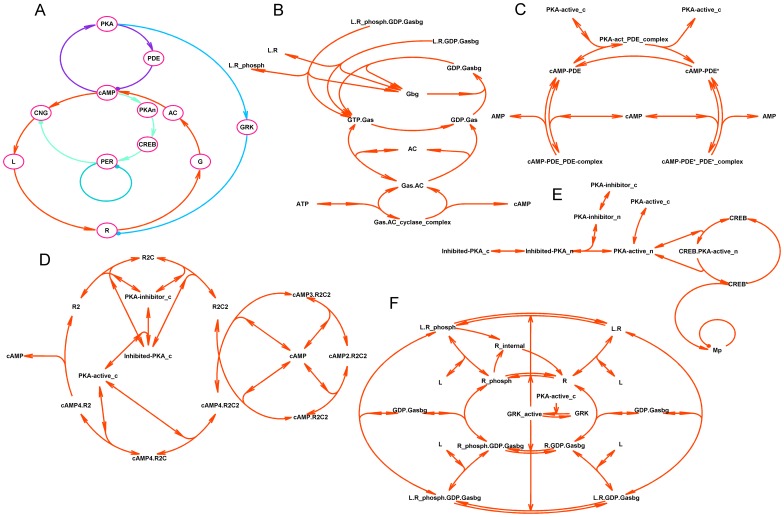
Schematic representation of the model of fast oscillations of firing rate (FOFR) in rat suprachiasmatic nucleus neurons cultured in multielectrode array dish. **A.** A simplified representation of key molecular interactions in the model. A positive and main negative feedback loops are shown in red and blue, respectively. PKA-PDE loop is depicted in violet. Interactions of cytoplasmic oscillator with a nuclear circadian oscillator (dark green) are shown in light green. cAMP – cyclic AMP, CNG – cyclic nucleotide gated channels, L – VIP, R – VPAC_2_ receptors, G – Gαs subunit of G protein coupled to VPAC_2_, AC – adenylate cyclase, PKA – protein kinase A, PDE – phosphodiesterase, GRK – GPCR coupled kinase, PKAn – nuclear PKA, CREB - transcription factor CREB, PER – Period gene mRNA. **B-F.** A detailed schematic description of the molecular interactions modeled (see Text S1 for details). A proposed mechanism of autocrine control of observed 30-min oscillations of firing rate suggests that the binding of external VIP (L) to VPAC_2_ receptor (R) activates G_s_ protein (Fig. 1F). The activated α-subunit of G_s_ protein dissociates from its respective βγ-subunits, and activates the production of cAMP by adenylate cyclase (AC) (Fig. 1B). Cyclic AMP activates cation CNG-channels, which depolarize the SCN neurons (Fig. 1A). Depolarization of model neuron evokes action-potential (AP) firing that, in turn, induces VIP secretion (Fig. 1A). This sequence provides positive feedback loop for the mechanism of FOFR. Simultaneously, cascades of events interrupting the positive feedback loop are present in our model. First, four cAMP molecules sequentially bind to each of protein kinase A (PKA) receptor subunits leading to release of two activated catalytic subunits of PKA (Fig. 1D). Then, PKA activates cAMP phosphodiesterase (PDE), which transforms cAMP to AMP (Fig. 1C). Second, the same PKA evokes desensitization and internalization of VPAC_2_ receptors via the phosphorylation of G protein-coupled receptor kinase (GRK) (Fig. 1F). FOFR-generating signaling cascades interact with nuclear circadian oscillator though PKA/CREB/Per signaling cascade (Fig. 1E).

In this work, we use (i) molecular processes originally modeled by Bhalla [18] describing the VIP-activated cAMP/PKA signal transduction [19], (ii) experimental data demonstrating functioning of cyclic nucleotide-gated (CNG) channels in SCN neurons [20], [21], and (iii) experimental data that characterizes VPAC_2_ receptor desensitization and internalization [22]–[25]. Signaling cascades described in the model are shown schematically in Fig. 1. A detailed description of the model can be found in Appendix (Text S1). Equations were integrated using MATLAB ode15s solver and analyzed using MatCont software [26].

We summarized the key data collected in experiments into the following model assumptions:

1. VIP signaling is crucial for the synchronization of circadian oscillations in the SCN neuronal network [12], [27]–[29]. We suppose that the same auto/paracrine signaling cascades are involved in both FOFR in a single neuron (Fig. 1A) and synchronization of FOFR in a network of heterogeneous SCN neurons. For simplicity, it is suggested that a direct synaptic or gap-junctional coupling between oscillatory SCN neurons is not essential for the synchronization. In accordance with our suggestion, firing rate-dependent release of VIP (ligand, L, in Fig. 1A) in an extracellular milieu activates G-protein coupled VPAC_2_ receptors (R in Fig. 1A) of the same neuron [28].
2. We used a model of VIP signaling in SCN neuron [19] as a core of our model. This core model simulated VIP-induced VPAC_2_ receptor activation and the resulting, activation of adenylate cyclase (AC in Fig. 1A, Fig. 1B) by VPAC_2_ receptor-associated G_s_ subunit (G in Fig. 1A, Fig. 1B) [30]–[32]. It has been shown that acutely isolated SCN neurons express cation CNG channels [21], [33]. Their activation, subsequent depolarization of the plasma membrane and action-potential (AP) firing [21], [33] followed by VIP release finalized positive feedback loop in our model (red arrowhead circle in Fig. 1A). Thus, the core model was completed by adding equations describing activation of cation CNG-channels by cAMP, subsequent membrane depolarization and AP firing, and VIP secretion (Eqs 44, 46–52 in Text S1).
3. An additional negative feedback loop capable of breaking down the positive feedback loop at high VIP levels is crucial for the generation of oscillations. We hypothesized that well-characterized phenomenon of desensitization and internalization of VPAC2 receptors [22]–[25] could provide an appropriate negative feedback in this system. Desensitization occurrs due to phosphorylation of VPAC_2_ receptor by G protein-coupled receptor kinase (GRK in Fig. 1A, Fig. 1C–1F), and activity of GRK in VPAC_2_-expressing cells is regulated by protein kinase A (PKA in Fig. 1A, Fig.1D) phosphorylation [25]. As a result of this phosphorylation, a dose-response curve of VPAC_2_ activity is right-shifted [25] and fast removal of receptors from cytoplasmic membrane surface takes place. The rate of recovery of plasma membrane VPAC_2_ receptors after their internalization [22], [23], [25] is the slowest rate constant in our model and thus, this rate constant is the main determinant of the period of FOFR. Notably, inverse of this rate constant representing the characteristic time of VPAC_2_ receptors recovery is comparable with the ∼30-min period of FOFR that were observed in cultures of SCN neurons [15]. This fact speaks in favor of the assumption that VPAC_2_ internalization may play role in the generation of FOFR. Equations that describe activation of GRK2 by PKA and phosphorylation of VPAC_2_ receptors by GRK2 (Eqs. 37–42 in Text S1) were derived from experimental data [23]–[25]. Namely, the model was tuned to match experimentally observed rates of desensitization and internalization [23]–[25], rates of recovery from desensitization and internalization [23]–[25], dependence of GRK activity on the concentration of active PKA, and dependence of cAMP concentration on VIP concentration before and after desensitization and internalization of VPAC_2_ receptors [25]. Equations describing VPAC_2_ receptors internalization were modified from Hao et al [19] in order to obtain an appropriate description of experimentally observed properties of this process (Eqs. 17, 37, 39, 45 in Text S1) and supplemented with an equation describing 20–40 min time course of recovery of internalized plasma membrane VPAC_2_ receptors (Eq. 45 in Text S1).

### Modeling the circadian synchronization in the SCN network with FOFR

To study the effect of FOFR on synchronization of circadian rhythms in the SCN neuronal network we incorporated a model of the mammalian circadian clock developed by Leloup and Goldbeter [34] into our model of SCN neuron developed to reproduce ∼30-min oscillations of firing rate. The interaction of VPAC_2_ receptors with circadian oscillations was described by introducing an additional term into the Eq.1 of Leloup and Goldbeter's model [34] (see also Eq.1 in [19]) which described dependence of Per gene expression on the CREB activity (see Text S1, Eq. 36, for details):
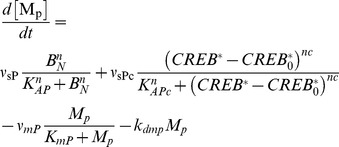
(1)


In this work we considered three models of circadian regulation of SCN neurons firing (Eqs. 49–52 in Text S1):

1. **Model without VIP/CNG coupling.** A firing rate of SCN neurons is regulated exclusively via activation of CNG-channels by Per gene product, i.e., VPAC_2_ receptors do not activate CNG channels via the above described G_s_/AC/cAMP pathway (the percentage of CNG channels that are independent of local cAMP level,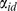 in Eq 51 from Text S1, was set to 1; see “Modifications of the default parameter set” section in Text S1). In this case the model of To et al [14] was used with an addition of hypothetical cascade between Per mRNA and the extracellular VIP.
2. **Model with VIP/CNG coupling with FOFR.** The firing rate is regulated by two pools of CNG channels. In this model most of the CNG channels (80%) are coupled to VPAC_2_ receptors via G_s_/AC/cAMP pathway and functioning of these channels do not depend on circadian clock molecular signals (Eq. 50 from Text S1); the minority (20%) of CNG channels are regulated by Per gene expression in a fashion similar to that described for the Model 1 above (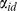 in Eq. 51 from Text S1 was set to 0.2).
3. **Model with VIP/CNG coupling without FOFR.** The firing rate is regulated by two pools of CNG channels as in the Model 2 (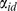 was set to 0.1), but VPAC_2_ receptors desensitization was removed by setting an initial GRK level to zero. In addition, parameters of the model were modified in such a way that its dynamics was close to the transition from monostability to bistability (see “Modifications of the default parameter set” section in Text S1). These settings created conditions for the efficient amplification and averaging of circadian nucleus-to-membrane signal without induction of FOFR.

The degree of the circadian activity synchronization was estimated in two different ways: first, as a standard deviation of either phases of circadian oscillations of firing rate or Per gene product concentration in a population of neurons and second, by introducing the synchronization index (SI) [35]:
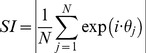
(2)


The value of SI ranged from 0 (no synchronization) to 1. For each cell, the phases 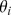 were calculated as positions of the center of mass of the cell activity during the successive 24-h periods.

## Results

### Modeling of VIP-induced cAMP accumulation and internalization of VPAC_2_ receptors

VPAC_2_ is a Gs protein-coupled receptor activated by the endogenous peptide VIP. As VPAC_2_ activates the adenylate cyclase (AC) - cAMP signaling pathways [36], simulation of VIP application should produce an increase of cAMP concentration in the cytosol.

**Figure 2:**
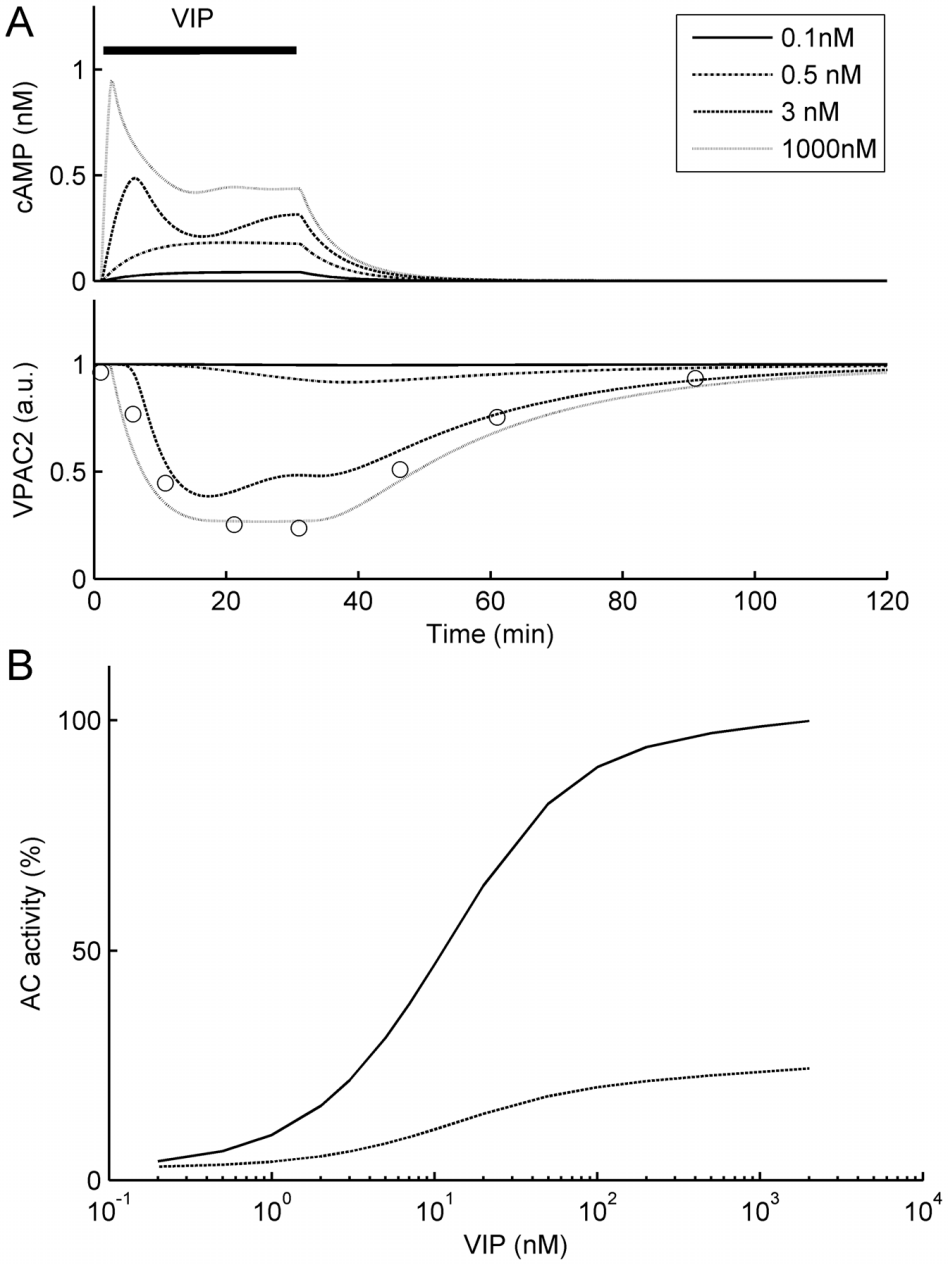
Basic properties of VIP-cAMP signaling in the model of FOFR. **A**. Response of the model neuron to the application of different VIP concentration steps (from 0.1 nM to 1 µM). Shown are concentration of cAMP (nM) and the ratio of the number of membrane VPAC_2_ receptors to the total number of VPAC_2_ receptors. For comparison, experimental data from Murthy et al. (Figure 10 in [25]) describing ^125^I-VIP binding to surface VPAC_2_ receptors during and after VIP (1 µM) application are shown by open circles. **B**. Dependencies of adenylate cyclase (AC) activity (measured as % of maximal concentration of G_αs_-AC complexes) on VIP level before (smooth line) and after (dotted line) desensitization and internalization of VPAC_2_ receptors induced by the application of VIP (1 µM) for 30 min.

For low VIP concentrations (<∼1 nM), the time course of simulated cAMP accumulation represented an exponential rise to some steady-state level, while for higher concentrations (>∼1 nM) this accumulation demonstrated a transient peak within 5–10 min after the onset of VIP application followed by a slow decrease to a steady-state level. In both cases, the subsequent washout of VIP returned cAMP concentration to a basal level (Fig. 2A, upper plot). Simultaneously, in both cases simulated VIP treatment resulted in slowly developing internalization of VPAC_2_ receptors reaching a plateau (Fig. 2A, lower plot). Washout of VIP resulted in recovery of the VPAC_2_ receptors concentration in the plasma membrane to a basal level. A half-time of VPAC_2_ receptors internalization at 1000 nM of VIP was ∼4.6 min, and the number of receptors in the plasma membrane reached 85% of its initial value within 1 h after VIP washout. The maximal level of internalization VIP was 76%, and the recovery half-time was ∼20 min in this case. Obviously, a transient peak with a subsequent plateau in cAMP concentration was due to internalization of VPAC_2_ receptors, forming in this manner a negative feedback, which stabilized intracellular cAMP concentration. It is noteworthy that the results of these simulations were in agreement with experimental data (Fig. 10 in [25]).

We have also modeled the dependence of AC activity, estimated as a concentration of AC bound with G_s_ subunit, on the VIP concentration before and after VPAC_2_ receptors desensitization and internalization. To do this we have simulated 30 min application of 1 µM of VIP. Then, after simulated 5-min washout, the AC activity in a response to the application of different testing VIP concentrations was calculated and plotted versus these testing VIP concentrations. AC activity was measured in two minutes after VIP application and was expressed as a percent of the highest possible concentration of G_αs_-AC complexes. Figure 2B demonstrates that the prolonged activation of VPAC_2_ receptors resulted in ∼4-fold decrease in the maximal value of AC activity without a significant shift of AC half-maximal activity. The observed dose-dependence curves (Fig. 2B) were also similar to the experimental data (Fig. 13 in Murthy et al. [25]) indicating the values of experimentally measurable parameters produced by our model are close to experimental data.

### A Model of FOFR in the single cell

Mechanisms producing periodical cAMP oscillations have been studied and modeled in many cellular systems [17], [37]–[39]. In the presented model, oscillations of external VIP concentration as well as oscillations of other parameters controlling its release, e.g. electrical firing rate, are expected to be observed due to the existence of the positive autocrine effect of VIP. Indeed, our simulations demonstrated that the model exhibited oscillations of these and other parameters with a period that fall within the range of experimentally observed values for the period of FOFR in cultured SCN neurons (20–60 min, [15]).

**Figure 3:**
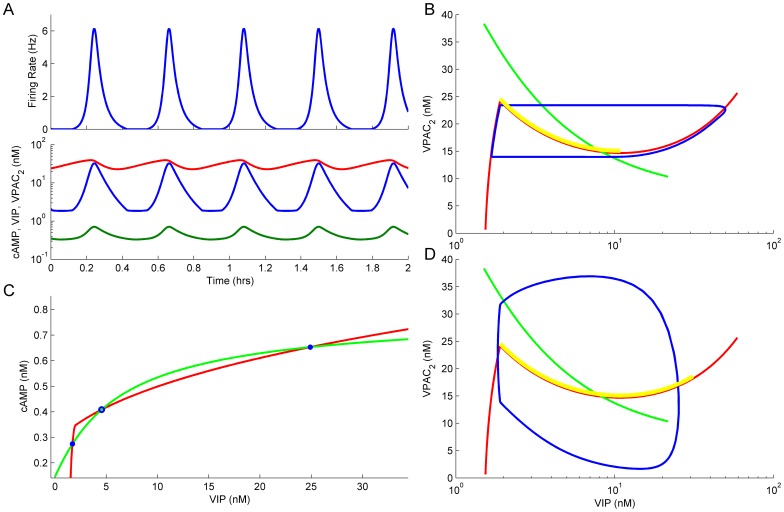
Mechanism of oscillatory activity in the model. **A**. Firing rate and concentration of the key molecular players during FOFR. **B**. Dynamics of the model in the VIP – VPAC_2_ plane in the case of a very slow rate of recovery of membrane VPAC_2_ receptor concentration after internalization of these receptors (k_71_ was set to 10^−8^ s^−1^, see Text S1). VPAC_2_ nullcline is shown in green, VIP nullcline is shown in red. A stable limit cycle is shown in blue. The range of the nullclines intersection in which stable oscillations were observed is shown in yellow. **C**. Dynamics of the model in the VIP – cAMP plane under fixed VPAC_2_ membrane concentration. VIP nullcline – green, cAMP nullcline – red, stable equilibrium – closed circles, unstable equilibrium – open circle. **D**. Dynamics of the model in the VIP – VPAC_2_ plane with experimentally observed rate of recovery from receptors internalization. All notations are the same as in B.

An example of such oscillations for internal cAMP and external VIP concentrations as well as concentration of membrane VPAC_2_ receptors and firing rate (model parameters are described in Table 1 in Text S1) is shown in Fig. 3A. A proposed mechanism of autocrine control of observed 30-min oscillations of firing rate suggests that binding of external VIP to VPAC_2_ receptor activates G_s_ protein (Fig. 1F). The activated α-subunit of G_s_ protein dissociates from its respective βγ-subunits and activates the production of cAMP by adenylyl cyclase (AC) (Fig. 1B). cAMP activates cation CNG channels, which depolarize the SCN neurons (Fig. 1A). Depolarization of model neuron evokes action-potential (AP) firing that, in turn, induces VIP secretion (Fig. 1A). This sequence provided a positive feedback loop for the mechanism of 30-min oscillations. Simultaneously, cascades of events interrupting the positive feedback loop were incorporated into our model. First, four cAMP molecules sequentially bind to each protein kinase A (PKA) receptor subunits and release two activated catalytic subunits (Fig. 1D). Then, the PKA activates cAMP phosphodiesterase (PDE), which transforms cAMP to AMP (Fig. 1C). Second, the same PKA evokes desensitization and internalization of VPAC_2_ receptors via phosphorylation of G protein-coupled receptor kinase (GRK) (Fig. 1F). Recovery from desensitization and internalization, which are the slowest processes in the model, determines the 30-min period of firing rate oscillations.

Analysis of the oscillatory mechanism in the system of 47 equations is complicated, but it can be simplified taking into account that the recovery from desensitization and internalization of VPAC_2_ receptors are the slowest processes in the system. In the limit case of very slow rate of receptors internalization, evolution of the system on the VIP - VPAC_2_ plane takes place along an orbit lying close to the VIP nullcline (i.e. a line of equilibrium VIP concentration for a fixed number of active VPAC_2_ receptors). This nullcline has a characteristic N-shape (a red line in Fig. 3B), which reflects the fact that if the number of VPAC_2_ receptors in the system is fixed then the system has a region of bistability. This bistability arises as a property of VIP-cAMP positive feedback loop: at low VIP and cAMP levels this feedback does not work - both variables evolved rather independently and their concentrations remained low; at higher levels strong positive interaction raised concentrations of both molecules closely to some high stationary levels restricted by the saturation rate of cAMP production. These interactions are illustrated in Fig. 3C, where nullclines of the system with a fixed number of VPAC_2_ receptors are shown in the cAMP – VIP plane. These nullclines have two or three points of intersection depending on the number of VPAC_2_ receptor molecules in the plasma membrane. In the particular case shown in Fig. 3C there are 3 points of intersection and thus, the system has three fixed points two of which are stable (shown by filled circles). In the ideal case of slow receptor internalization, relaxation oscillations would arise in the system when the VPAC_2_ nullcline (a green line in Fig. 3B) intersects the VIP nullcline (a red line in Fig 3B) in its descending phase (i.e., along the region shown in yellow in Fig. 3B). In this case, the system evolves repeatedly along the two positive slope regions of the VIP nullcline with rapid transitions between them (blue line in Fig. 3B). For experimentally observed rates of receptor desensitization and internalization, the set of intersection points between the nullclines that yield stable oscillations was estimated by continuous shifting of the VPAC_2_ nullcline (green line in Fig. 3D) to the right and monitoring the oscillation amplitude. Shifting of the VPAC_2_ nullcline was implemented by changing initial concentration of GRK in the model. As it can be seen from Fig. 3D, the obtained set of intersection points (yellow region in Fig. 3D) mostly coincided with the descending phase of the VIP nullcline. The stable limit cycle also arised in this case and it is shown in Fig. 3D (blue line).

### Sensitivity analysis of model parameters

To assess the robustness of oscillations with respect to the variation of model parameters, we performed two series of computational experiments. Firstly, we systematically varied initial concentrations of all molecules in order to define the value range in which oscillations of cAMP concentration with amplitude higher than 10% of average cAMP level would be observed for longer than 15 hours of model evolution. Secondly, we defined a range of all kinetic rates and parameters added to Hao's et al model [19] in which oscillations with the above mentioned characteristics occured. The varied parameters were: the rates for activation of GRK by PKA_active (k_61_), dephosphorylation of GRK (k_62_), phosphorylation of VPAC_2_ by GRK (k_63_), dephosphorylation of VPAC2 (k_64_), phosphorylated VPAC_2_ receptor internalization (k_42_), insertion of new VPAC2 receptors (k_70_). The other varied parameters were: maximal conductivity of CNG channels (g_CNG, 0_), steady state VIP concentration (VIP_0_), threshold for AP generation (I_threshold_) and the ratio of the rates of binding with Gs of phosphorylated to dephosphorylated VPAC_2_ receptors (DE).

**Figure 4:**
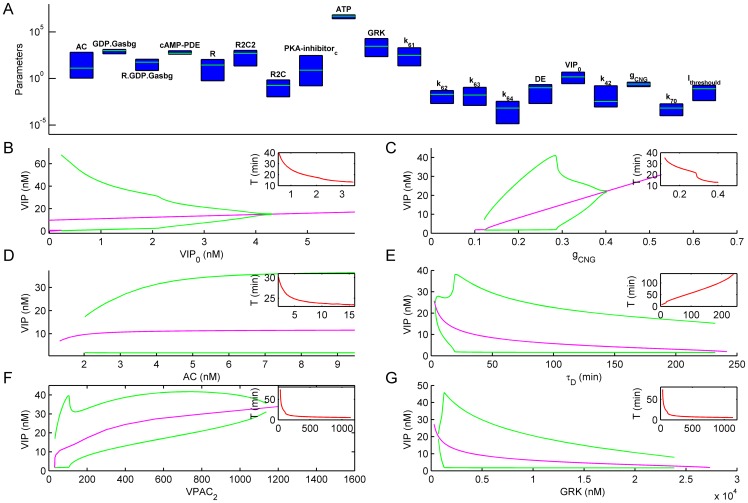
Parameter sensitivity of the FOFR model. **A**. The ranges of parameters for which oscillations were observed. Initial concentrations of all molecules in the model, a key kinetic rates and some parameters from those that we have added to the model of Hao et al. [19], were sequentially varied in the range [x0/50, x0*50] (where x0 is default initial concentration). 15 hours of the model state evolution was simulated, and the parameter ranges were found in which the oscillations of cAMP concentration with the amplitude of more than 10% of the cAMP average level was observed. Only those parameter ranges (blue bars) are shown for which oscillations disappeared during the parameter variation in a range smaller than [x0/50, x0*50], where x0 is default initial value of each parameter (shown in green, scale is logarithmic). **B-G**. Bifurcation diagrams of the equilibrium (magenta line) for a key model parameters. In addition to the equilibrium, the range of stable limit cycle is shown (green lines). Dependencies of the period of oscillations on each of the model parameters are shown in the inset.

Results of these computational experiments are presented in Fig. 4A. Only for 10 out of 47 molecules described in the model variations of their initial concentrations in the range 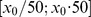 (where 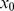 is the default initial value) resulted in disappearance of oscillations. These were AC, Receptor-G-protein complex (R.GDP.G_asbg_), GDP bound Gs trimeric complex (GDP.G_asbg_), cAMP phosphodiesterase (cAMP-PDE), VPAC_2_ Receptors (R), PKA heterotetramer with two molecules of regulatory subunits and two molecules of catalytic subunits (R2C2), PKA heterotetramer reforming intermediate (R_2_C), Cytoplasmic PKA inhibitor (PKA-inhibitor_C_) and ATP. Parameter ranges (blue bars; Fig. 4A) in which oscillations were preserved during variation of parameters are shown in a logarithmic scale for the 10 most critical initial concentrations described above and for all added parameters. The oscillations were most sensitive to the following parameters (listed here in order of their influence on the oscillations): concentrations of PDE, ATP and G_βγ_ and conductance of CNG channels. For these parameters, the ratio of upper to lower boundaries of their values at which oscillations were still observed was 2.4, 2.6, 3.0 and 3.15, respectively. At the same time, this ratio was larger than 19 for other parameters. Thus, FOFR could be observed in our model for a wide range of model parameters, indicating model robustness.

To study in detail the parameter dependence of system dynamics, we have calculated positions of fixed points and stable limit cycles in the system while changing 6 most critical model parameters. Figure 4 shows dependencies of equilibrium positions (a red line) on concentrations of the key molecular players – VIP_0_ (Fig. 4B), CNG-channels (Fig. 4C), AC (Fig. 4D), VPAC_2_ (Fig. 4F), and GRK (Fig. 4G), as well as characteristic time of recovery from internalization for VPAC_2_ receptors, τ_D_ (Fig. 4E; τ_D_ was set to an inverse rate of recovery from internalization, k_70_). Fig. 4 also shows amplitudes of stable limit cycle (green lines). Dependencies of the oscillation periods on each model parameter are shown in the insets. All the bifurcation diagrams have a characteristic structure: when VIP-induced depolarization exceeds a firing threshold, oscillations appear abruptly having low frequency and high relative amplitude. Subsequently, they disappear in a fashion observed in the canard explosion scenario [40]: a fast transition from relaxation oscillations to relatively high frequency low amplitude oscillations. It can be concluded from these bifurcation diagrams that the oscillations with properties that fell within the experimentally observed range could be observed for a wide range of the key model parameters. The parameters differ in their potency to influence the period of oscillations. As it can be predicted, the period of oscillations is determined by the time course of receptors internalization (Fig. 4E), but it is also strongly correlated with the number of VPAC_2_ receptors (Fig. 4F) and the concentration of GRK (Fig. 4G).

### A model of FOFR in a heterogeneous cell population

An important property of FOFR observed in dispersed and cultured SCN neurons, which we expected to have a physiological significance, was synchronization of electrical activity between cells located in distant parts of the 1-mm recording area of a multielectrode array dish [15]. In many cases, such synchronization of FOFR was observed without obvious correlation of AP firing in studied neurons. This fact allowed us to exclude a principal role of excitatory synaptic connections in this phenomenon and to suggest that synchronization is provided by diffusible VIP in the intercellular milieu [14], [41]. In order to investigate whether a heterogeneous population of model neurons could oscillate in synchrony, we developed a model of a neuronal network with substantial random scattering of initial parameters between individual neurons. Initially, a short period of neurons behavior was simulated supposing that there is no VIP exchange between them. In this case each cell sensed only autocrine VIP. After this short period VIP exchange between the neurons within the network was allowed, i.e. VIP concentration sensed by all the cells in the network was set to the same value so that the cells could oscillate in synchrony.

**Figure 5:**
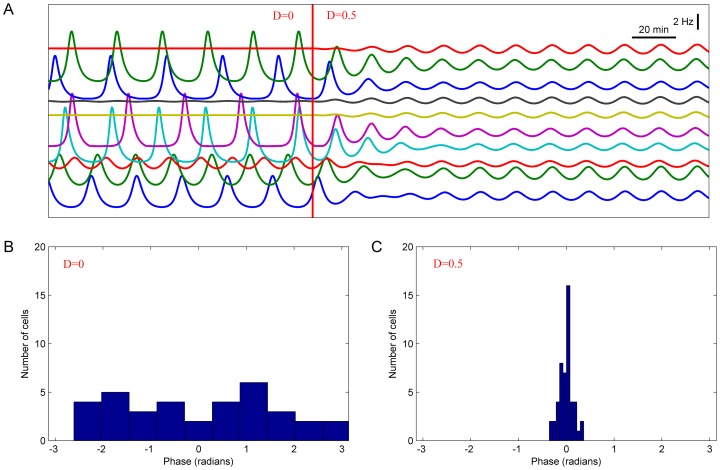
Synchronization of the oscillations in heterogeneous population of SCN neurons. The model included 50 SCN neurons that initially oscillated independently. Initial concentration of each of the molecular species in the model was selected randomly from the interval [x0−0.2*x0; x0+0.2*x0], and each rate constant – from the interval [k0−0.2*k0; k0+0.2*k0], where x0 and k0 are default values for initial concentration and kinetic rate for each molecule and chemical transition in the model, respectively. **A**. Firing rates for 10 of 50 SCN neurons in the population before and after enabling VIP exchange between the neurons. A vertical red line marks the time when intercellular exchange by VIP was switched on (D = 0.5, Figs. 5A, 7A_1_, B_1_, C_1_) and off (D = 0, Figs. 5A, 7A_2_, B_2_, C_2_). **B, C**. Distribution of oscillation phases before (B) and after (C) introduction of VIP exchange.

A typical example of model experiment that was conducted with a network of interacting SCN neurons is shown in Fig. 5. The model included 50 cells that oscillated independently until VIP exchange was enabled. Each initial concentration in the model was randomly selected from an interval 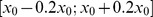, and each rate constant was also randomly selected from an interval 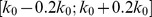, where 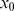 and 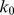 are default values for initial concentrations and kinetic rates for each molecule and chemical transition in the model (see Text S1). Oscillations were initially observed in 70% of the cells (35 of 50). A period of oscillations varied from 14 min to 36 min (mean±SD = 23.0±5.3 min) and their amplitudes varied from 0.1 Hz to 9 Hz (4.8±2.3 Hz). Examples of firing rate changes for silent and spontaneously active cells are shown on the left from a vertical red line in Fig. 5A, and the respective distribution of oscillation phases is shown in Fig. 5B. After 20 hours of the independent evolution of each cell (D = 0), VIP exchange was enabled (D = 0.5 after a vertical red line in Fig. 5A). As a result, oscillations of different neurons in the network became highly synchronized and a distribution of their amplitudes gradually became narrower (Fig. 5A, to the right of the vertical red line, and C). The oscillation period varied in different cells in a very narrow range from 16.7 min to 16.9 min (16.77±0.06 min) and the range of the respective oscillation amplitudes was from 1.2 Hz to 3.1 Hz (1.75±0.45 Hz). Distribution of oscillation phases was markedly narrowed as a result of neuronal network synchronization (standard deviation 24 sec).

**Figure 6:**
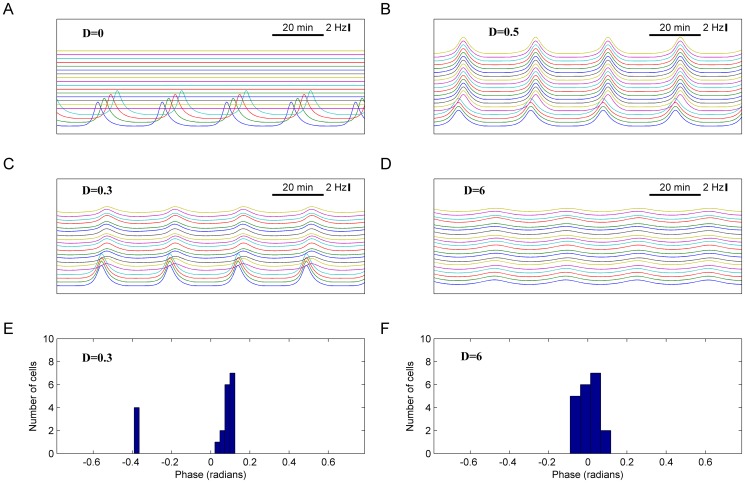
Induction of the oscillations in the heterogeneous population of SCN neurons by a small group of oscillating cells. Network of 20 cells was modeled, with 4 cells having default parameter set and other 16 cells having CNG conductance twice lower than default and all other parameters as default. **A**. Firing rate in the population before introduction of exchange by VIP. Only 4 abovementioned cells generate oscillations of firing rate. **B**. Firing rate in the population after the exchange by VIP was introduced (D = 0.5). All 20 cells started to oscillate in synchrony. This result was robust with respect to CNG channel conductance and moderate random perturbations of parameters. 4 cells with default parameters were capable to induce synchronous oscillations in the remaining 16 cells with reduced (20% of default value) CNG channel conductance. All other parameters of the model were selected for each of these 16 cells randomly and uniformly from the interval [p0-0.05*p0; p0+0.05*p0], where p0 is default value of each parameter. **C, E**. Firing rate and phase distribution in the population of SCN neurons after introduction of weak coupling through VIP exchange (D = 0.3). **D, F**. The same plots for strongly coupled oscillators (D = 6).

As was shown in [9], [42], [43], less than 20% of SCN cells exhibit circadian firing rate oscillations in low-density cultures, while almost all cells oscillate when cultured at high density. Therefore, we further addressed a similar question of whether the 20% of cells that exhibited FOFR evoked by autocrine VIP activation without VIP exchange could evoke persistent synchronous oscillations in other cells when intercellular exchange of VIP was enabled. In this set of experiments a network of 20 cells was modeled, with 4 cells having default parameters (see Text S1) and other 16 cells having CNG conductance twice lower than default, while all other parameters were set to their default values. Initially there was no intercellular VIP exchange, and only these 4 cells generated oscillations of firing rate (Fig. 6A, D = 0). When VIP exchange was enabled (D = 0.5), all 20 cells started to oscillate synchronously (Fig. 6B) with a mean period of 28.19±0.01 min, amplitude of 4.20±0.03 Hz, phase SD of 47 sec. Thus, our modeling indicates that VIP exchange in the SCN network having 20% of intrinsically oscillating neurons results in the synchronous oscillations of entire neuronal network. This result was robust with respect to both CNG-channel conductance and moderate random perturbations of parameters. Figures 6C, D demonstrate that 4 cells having default parameters were capable to induce synchronous oscillations in the other 16 cells having 20% of default CNG conductivity while all other parameters of the model were selected for each of these 16 cells randomly and uniformly from an interval 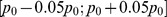 where 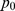 is a default parameter value. It is known [44] that the amplitude of network oscillations inversely depends on the strength of coupling between oscillators (so called oscillation death phenomenon). Correspondingly, in our model increase of coupling strength resulted in expected rise of synchrony while the amplitude of FOFR decreased. For weakly coupled oscillators (D = 0.3, Fig. 6C, E) the phase SD was 49 sec, amplitude mean±SD – 2.5±1.5 Hz, period of oscillations – 26.6 min. For strongly coupled oscillators (D = 6, Fig. 6D, F), the degree of synchrony was characterized by phase SD of 18 sec, amplitude mean±SD of 1.0±0.1 Hz, period of oscillations – 27.77±0.03 min.

### A model of the circadian rhythm synchronization in the SCN network with FOFR

In accordance with our conception, existence of synchronous FOFR in a cultured network of SCN neurons suggests that their firing activity can be promptly and strongly coupled to the activation of VPAC_2_ receptors. Such coupling may be important for the synchronization of the circadian activity of SCN network, since the phase of circadian electrical activity for each SCN neuron could be determined in this case by the VIP level in extracellular milieu rather than exclusively from the gene expression pathway [14], [35]. In order to study the importance of this coupling in a computational experiment, we added the model of the mammalian circadian clock developed by Leloup and Goldbeter [34] to our model of the SCN neuronal network with FOFR. The interaction of VPAC_2_ receptors with a circadian genes network was modeled as described in Materials and Methods (Fig. 1). We considered three models of circadian regulation of neuronal firing (see ‘Modeling the circadian synchronization in the SCN network with FOFR’ in Materials and Methods).

Two series of experiments with 10-cell networks were conducted to demonstrate that VPAC_2_/CNG-channel coupling in the SCN network could help to achieve rapid and precise synchronization of electrical firing activity even if synchronization of circadian genes expression is slow and imperfect.

**Figure 7:**
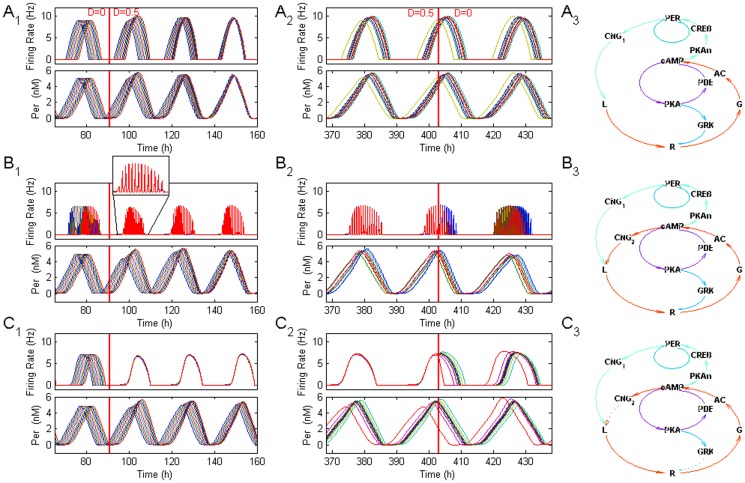
Colocalization of VPAC_2_ receptors and CNG channels improve synchronization of the circadian activity in the network of SCN neurons. A_1_. Firing rates (top) and Per gene expression (bottom) before and after introduction of VIP exchange (red vertical line) in the network of 10 cells. The firing rate was regulated directly by expression of circadian genes (Model without VIP/CNG coupling, 1^st^ experiment, see Methods). All cells had default sets of parameters in both circadian clock and FOFR models. Circadian discharges started with phase shifts uniformly distributed within 6 h interval and without exchange by VIP (D = 0) up to the 91 hour (red vertical line). Then VIP exchange between cells was introduced (D = 0.5). A_2_. The same variables after stabilization of circadian oscillations of firing rate and Per gene expression in heterogeneous population of coupled oscillators before (D = 0.5) and after (D = 0) their uncoupling (Model without VIP/CNG, 2^nd^ experiment). All cells had default sets of parameters of fast oscillations but circadian clock parameters were randomly and uniformly perturbed within 3.5% interval around their default values. A_3_. Scheme of the respective Model without VIP/CNG coupling. B_1_, B_2_. The results of the same experiments as in A_1_, A_2_ for the Model with VIP/CNG coupling with FOFR, i.e. when firing rate was mostly regulated by external VIP via CNG channels colocalized with VPAC_2_ receptors, but with influence of Per gene product on the conductance of the minor fraction of CNG channels (see Methods). B_3_ Scheme of the respective Model with VIP/CNG coupling with FOFR. C_1_, C_2_. The same experiments as in A, B, for the Model with VIP/CNG coupling without FOFR (i.e. when firing rate was influenced by both fast and slow VIP signaling loops similar to the Model with VIP/CNG coupling with FOFR, but there were no FOFR, see Methods) C_3_. Scheme of the respective Model with VIP/CNG coupling without FOFR.

In the first series of experiments, all cells had default parameters for both circadian clock and FOFR models. Circadian oscillations in each cell had a uniformly distributed phase shift. Initially, cells oscillated independently (D = 0) up to the 91 hour, when coupling between oscillators was introduced (D = 0.5). The typical results of such experiments are shown in Fig. 7, where each row represents a different model of circadian regulation of firing activity.

#### Model without VIP/CNG coupling

Firing rate was (indirectly) regulated exclusively via activation of CNG-channels by Per gene product as it is shown schematically in Fig. 7A_3_. This model is similar to To's et al model [14] but additionally contains a hypothetical cascade that detailed interaction between Per mRNA and the extracellular VIP. The synchronization of firing rates in different SCN neurons developed slowly (Fig. 7A_1_, vertical red line indicates coupling switch-on) and its time course was comparable to the time course of synchronization of Per gene expression in different SCN neurons. Indeed, SD of phase of circadian firing rate oscillations before coupling was 2.9 h, whereas it was 3.1 h (106%) and 1.5 h (52%) at 24 h and 72 h after enabling of the coupling. The respective values for SD of oscillation phase of Per gene expression were 2.4 h, 2.5 h (104%) and 0.51 h (21%), respectively.

#### Model with VIP/CNG coupling with FOFR (Fig. 7B_3_)

The firing rate of SCN neurons was mainly regulated by the external VIP concentration with certain influence of Per gene product on the conductivity of the minor part of CNG channels. There were two pools of CNG channels in the model: (i) most CNG-channels (80%) were coupled to VPAC_2_ receptors via G_s_/AC/cAMP pathway and their conductance was not dependent on circadian clock molecular signals; (ii) the rest of CNG-channels (20%) were regulated by Per gene expression by means of molecular mechanisms described in the above described model without VIP/CNG coupling. The synchronization of firing rates in these experiments appeared to be biphasic. The fast phase of the synchronization developed immediately after introduction of coupling (Fig. 7B_1_, red vertical line indicates coupling switch-on, see inset for synchronization) followed by slow phase of synchronization with a time course similar to the time course of synchronization of genetic circadian rhythms (right part of Fig. 7B_1_ with respect to the red vertical line). An initial SD of oscillation phase of firing rates was 1.4 h. It was abruptly changed to 0.26 h (18%) when coupling strength was switched on and was further reduced to 0.1 h (7%) in 72 h after enabling the coupling. At the same time development of Per gene expression synchronization was not significantly changed compared to the results obtained for the first model. E.g., SD of oscillation phase of Per gene expression was 1.1 h before coupling, 1.0 h (91%) immediately after the coupling and 0.42 h (38%) in 72 hours, respectively.

#### Model with VIP/CNG coupling without FOFR (Fig. 7C_3_)

In this model, parameters were chosen to create conditions for an efficient amplification and averaging of circadian nucleus-to-membrane signal without induction of FOFR itself. The firing rate was under control of both cytoplasmic VIP-CNG channels link and nuclear VIP-Per expression-CNG channels link as in the model with VIP/CNG coupling with FOFR, but fast oscillations were disabled. The results of experiments were similar to those obtained for the model with VIP/CNG coupling with FOFR: the degree of synchronization before coupling of network, after 24 h and 72 h, for firing rates was 2.6 h, 0.33 h (13%) and 0.18 h (7%), respectively and for Per gene expression: 2.4 h, 2.9 h (120%) and 1.8 h (71%), respectively (Fig 7C_1_). One could conclude that FOFR itself is not necessary for fast synchronization of circadian electrical peaks but the cytoplasmic G_s_/AC/cAMP/CNG-channels pathway could indeed accelerate synchronization of circadian electrical peaks in a neuronal network.

In the second series of experiments we have tested the dependence of a steady-state level of synchronization in heterogeneous cell populations on the model of circadian regulation of firing activity. All cells had default parameters of fast oscillations but circadian clock parameters were randomly distributed within 3.5% interval around their default values. Circadian oscillations started without a phase shift, and cells oscillated independently (D = 0) up to the 91 hour, then VIP exchange was introduced (D = 0.5) and oscillations become gradually synchronized. To estimate the contribution of CNG channels strongly coupled with VIP to the network firing synchronization, the VIP exchange was again set to zero (D = 0) after 403 hours of oscillations.

#### Model without VIP/CNG coupling

The degree of firing rate synchrony (SD of phase distribution) in heterogeneous network (Fig. 7A_2_) was 2.5 h after 400 h of oscillations. After coupling strength was set to zero (vertical red line in Fig. 7A_2_) SD was changed (24 hours later) to 2.7 h (106%).

#### Model with VIP/CNG coupling with FOFR

The degree of firing rate synchronization was 0.12 h after 400 h of oscillations, and it was changed abruptly to 0.60 h (520%) within 24 hours after coupling strength was set to zero (Fig. 7B_2_).

#### Model with VIP/CNG coupling without FOFR

The degree of firing rate synchronization was 0.18 h after 400 h of oscillations, and it was changed 24 hours later to 2.2 h (1210%) (Fig. 7C_2_). These results clearly show that degree of synchrony of circadian firing rate activity in heterogeneous population of SCN neurons could be increased significantly if the fast cytosolic signaling from VIP-receptors to the CNG channels is introduced.

Thus, it has been concluded that the phenomenon of fast coupling of VIP level with the firing rate underlying FOFR in the SCN network may provide a complementary mechanism for the synchronization of circadian firing activity in SCN, in addition to the synchronization of circadian electrical peaks through Per gene expression.

## Discussion

In this work we have presented a mathematical model of fast oscillations of firing rate with a typical period of about 30 min in both a single cell and neuronal SCN network that were earlier observed experimentally in dispersed and cultured neurons [15]. We have shown that incorporating the proposed mechanism of fast oscillations into a published model of synchronization of circadian peaks based on VIP-Per gene regulation [14] greatly accelerates synchronization of circadian firing rate oscillations.

### Model of FOFR in single neuron

The existence of FOFR in cultured SCN neuronal network suggests the existence of a strong and fast coupling between receptors for biologically active substance(s) and action potential generation ([15], Figure S1). This coupling previously was shown in direct electrophysiological experiments with SCN neurons in slice preparations [20]. In these experiments, application of VIP induced depolarization of neuronal membrane within several seconds. VIP is considered as a main paracrine signaling molecule in the SCN and it is known that its interaction with VPAC2 receptors on the neuronal surface induces elevation of cAMP concentration. Thus, the model developed in our work could be considered as a member of the class of biochemical oscillator models based on the cAMP signaling. Other examples of such models include oscillations of cAMP level in Dictyostelium discoideum amoebae [37], GnRH neurons of hypothalamus [17] and pancreatic insulin-releasing β cells [38], [39]. All these models have a similar structure with a positive feedback loop which consists of the G-protein coupled receptors for some paracrine substance, AC and some link between cAMP level and the rate of exocytosis of this substance. Another important component of these models is a negative feedback loop which breaks this positive feedback at high cAMP levels. This loop could be organized in a variety of ways. In GnRH neurons, this loop is organized via switching from Gs to Gi activity at high hormone levels [17], ]; in D. discoideum it is organized through blocking of AC by PKA [37] and desensitization of receptors for cAMP [45], [46] and in pancreatic cells it is organized through several mechanisms, including periodic activation of PDE by Ca2+-calmodulin kinase or PKA and calcium-dependent inhibition of AC [39]. In our model we have hypothesized that a negative feedback in a single SCN neuron is based on the well-known phenomenon of VPAC_2_ receptor desensitization and internalization [22]–[25]. We have shown that reversible internalization of receptors with properties that fall within the experimentally observed range is enough for the emergence of oscillations. However, we could not exclude that some other mechanisms not yet well characterized for SCN neurons, e.g., G_i_ and G_q_ subunit activation at high VIP levels, could also contribute to feedback generation [47], [48].

There were several reasons to introduce CNG channels in our model of FOFR. First, cAMP-signaling plays an important role in SCN pacemaking [39], [42]. Moreover, CNG-like channels were observed in acutely isolated SCN neurons [15], [49] and their activation by db-cGMP, membrane-permeable derivate of cGMP, resulted in the long-lasting firing [15]. Besides, an application of diltiazem, a blocker of CNG-channels, produced a reversible inhibition of firing in SCN neurons (N.I. Kononenko, unpublished data). Finally, cAMP with corresponding CNG-channels are key players of pulsatile electrical firing in a well-studied mechanism of 30-min oscillations in GnRH neurons [50]. In order to verify incorporation of CNG-channels in our model, one could study effects of their putative blockers on experimentally observed FOFR in MED.

We have also investigated the robustness of proposed mechanism of FOFR. The obtained results have shown that oscillations were highly robust with respect to the variations of all model parameters (Fig. 4). Therefore, the occurrence of FOFR in our model is a direct consequence of the model properties rather than of any specific choice of parameters.

### Synchronization of fast oscillations in neuronal network

It has been shown earlier that separate neurons in distant parts of the 1-mm MED recording area exhibited synchronized FOFR ([15], Figure S1). Our model successfully mimicked synchronization of 30-min oscillations of firing rate in the SCN network containing 20–50 cells (Fig. 5, 6). Notably, reliable synchronized FOFR in separate cells were observed when model parameters of each cell were randomly chosen in a broad range. Even if most of the cells were initially silent, as it is observed in dispersed and cultured SCN neurons [9], a small group of oscillating model neurons (∼20%) were capable to involve the silent cells into FOFR (Fig. 5, 6).

### Role of FOFR in synchronization of circadian firing peaks

The fast oscillations of electrical activity similar to modeled in this work have not been observed in the intact SCN yet. The reason for this could be the differences in tonic activity state and connectivity density between SCN cell culture, SCN in vitro and intact SCN, similar to the dependence of the amplitudes and other properties of globally synchronized oscillations of firing activity on the same parameters observed in cultures of neocortical neurons [51], [52]. Another possible explanation is that FOFR only occurs within a small subnetwork of the SCN. Nevertheless, the existence of these oscillations in the SCN neuronal cultures raises the possibility that the membrane potential of a particular SCN neuron during circadian oscillations can be controlled by the level of external neuromodulator(s) through coupling of their respective receptors and specific membrane conductance, rather than by the internal signal from the circadian genetic oscillator of the neuron, as it was assumed in other models [35], [53]–[55]. An intercellular exchange of these neuromediator(s) can efficiently amplify and average influences of the genetic oscillations of a large neuronal populations thus far inducing synchronous circadian oscillations of membrane potential in each neuron. In line with this assumption, recent experiments with SCN neurons cultured in MED have shown that synchronous FOFR arise together with synchronization of circadian peaks of electrical firing ([15], Fig. B, C, D1 and D2 in Figure S1). The synchronization of circadian electrical peaks in separate SCN cells is now considered to be regulated by Per gene expression only [14], but it is plausible to suggest that FOFR contributes to this process. Although this interpretation of experimental results ([15], Fig. D1 in Figure S1) should be done with caution, we attempted to model this putative process and found that FOFR could influence synchronization of circadian firing activity peaks.

We have shown that the Leloup-­Goldbeter model of circadian oscillators [34], with intercellular VIP exchange without FOFR, produces synchronization of circadian firing within 2.5–5 days after enabling VIP intercellular exchange (Fig. 7A1 and Fig. A in Figure S2). We have shown that the Leloup-Goldbeter model of circadian oscillators [8], with intercellular VIP exchange without FOFR, produces synchronization of circadian firing within 2.5–5 days after enabling VIP intercellular exchange (Fig. 7A1 and Fig. A in Figure S2). These results are close to those obtained in To's et al model [14] in which Per gene expression regulates VIP secretion. Incorporation of FOFR in the model including Leloup and Goldbeter circadian oscillator resulted in practically immediate synchronization of circadian peaks of electrical firing (Fig. 7B_1_, Fig. C in Figure S2). This effect can be easily observed in the model of SCN network with FOFR (Fig. 7 B_1_,B_2_, Fig. Fig. A, B in Figure S2) and still exists in the networks without FOFR although only for the narrow range of parameters (e.g., in the network near saddle-node bifurcation, Fig. 7 C_1_,C_2_, see ‘Modifications of the default parameter set’ and ‘Sensitivity of the synchronization of the circadian oscillations of firing rate to the model parameters’ in Text S1). Nevertheless, it may be assumed that even a small subnetwork of cells with FOFR can improve synchronization of circadian firing activity in the whole network. This assumption is in line with prediction that a small group of strongly connected circadian oscillators could underlie temporal precision of the electrical activity of the whole SCN network [43], [50]. Alternatively, synchronization can be achieved via slow signaling from the membrane to the genetic network in the neuronal nucleus and back to the membrane [35], [53]–[55]. It is natural to suggest that in this case the precision and rapidness of synchronization highly depends on the heterogeneity of signaling in these feedback loops and in circadian genetic networks in the population of neurons.

Several predictions based upon the FOFR mechanism's contribution to synchronization of circadian firing activity peaks can be experimentally tested: (i) reactivation of the paracrine signaling in the SCN network with partially desynchronized genetic oscillations should immediately improve synchronization; (ii) block of paracrine signaling [29] should reduce synchronization of circadian oscillations of firing activity in separate SCN neurons; (iii) synchronization of membrane potential oscillations should be stronger in comparison with synchronization of genetic networks. Experimental testing of these suggestions and further elaboration of the presented model of synchronization within SCN network are necessary for complete understanding of circadian clock mechanisms.

## Supporting Information

Text S1 **Appendix**: 1) System equations design and parameters fitting approach; 2) Chemical species used in the model; 3) Model Equations; 4) Parameter values; 5) Initial concentrations; 6) Modifications of the default parameter set; 7) Sensitivity of synchronization of the circadian oscillations of firing rate to the model parameters.
(DOC)

Figure S1 Fast oscillations of firing rate (FOFR) recorded in dispersed rat SCN neurons in multi-electrode array dishes (MEDs).
(DOC)

Figure S2 Synchronization of the circadian activity in the networks of SCN neurons with a random uniform distribution of initial phases of oscillation.
(DOC)
