# Supplementary material for: A Model for the Fast Synchronous Oscillations of Firing Rate in Rat Suprachiasmatic Nucleus Neurons Cultured in a Multielectrode Array Dish
Source: PLoS One. 2014 Sep 5;9(9):e106152. doi: 10.1371/journal.pone.0106152 (PMC4156468; doi:10.1371/journal.pone.0106152)
Supplement: Text S1 — Appendix: 1) System equations design and parameters fitting approach; 2) Chemical species used in the model; 3) Model Equations; 4) Parameter values; 5) Initial concentrations; 6) Modifications of the default parameter set; 7) Sensitivity of synchronization of the circadian oscillations of firing rate to the model parameters. (DOC) [file pone.0106152.s001.doc]

**APPENDIX**

**Mathematical model of 30-min oscillations in cultured SCN neuronal network**

1. **System equations design and parameter fitting approaches**

We used a model of VIP signaling in SCN neuron described in Hao et al [19] as a core of our model. The core model simulates VIP induced VPAC_2_ receptor activation. As result, G_s_ subunit (G in Fig. 1A) associated with VPAC_2_ receptor activates adenylate cyclase (AC in Fig. 1A) [30–32].

This core model (Eqs. 1-35) was complemented with the following equations:

(1) equations describing activation of cation CNG-channels by cAMP, subsequent membrane depolarization with corresponding AP firing, and VIP secretion (Eqs. 44, 46-52);

(2) equations describing activation of GRK2 by PKA, phosphorylation of VPAC_2_ receptors by GRK2 (Eqs. 37-42);

(3) equation describing internalization of VPAC_2_ receptors and recovery of internalized receptors on the membrane (Eq. 45).

In addition to this changes, equations from Hao et al [19] describing VPAC_2_ receptors internalization were modified to obtain appropriate description of experimentally observed properties of this process (Eqs. 17, 18, 11, 13, 15).

The interaction of VPAC_2_ receptors with circadian oscillations was implemented as additional term in Eq.1 of Leloup and Goldbeter’s model [34], which describes dependence of Per gene expression on CREB activity (Eq. 36).

All parameter values of the model were taken from [19] and [34] except for the parameters marked with bold letters in **Table S1** below**.**

The parameters describing dephosphorylation and internalization of VPAC_2_ receptors (k_42_, k_61_ - k_66_, k_70_, G) were taken directly from the works [24] and [25] or were obtained as result of the analysis of the data represented in these works.

Several parameters of the model (***Θ, ${\Delta L}_{\text{AP}}$, $L_{0}$,*** $g_{\text{CNG},0}$and concentrations of GRK and VPAC_2_ ) were fitted with a goal to obtain robust FOFR. First, cAMP and VIP nullclines (for a fixed concentration of VPAC_2_  receptors) were shifted at cAMP-VIP plane (Fig. 3C) to obtain their 3-point intersection. This guaranteed the existence of bistability in the system. The slope of cAMP nullcline was regulated by ${\Delta L}_{\text{AP}}$ and $g_{\text{CNG},0}$***,*** their position – by ***Θ*** and $L_{0}$***.*** The slope of VIP nullcline was tuned by changing VPAC_2_ concentration. Then, on the VPAC_2_  - VIP plane the slope of VPAC_2_ nullcline (green line in Fig. 3B) was regulated by changing GRK concentration until it intersected with a VIP nullcline (red line) in the region of negative slope of the latter. As result, system with robust FOFR was obtained.

When circadian oscillations in the network with FOFR were modeled, the ***KAP*** and $\nu_{\text{sPc}}$were the only fitted parameters in Eq. 36. The ***KAP*** parameter was fitted to obtain synchronization of circadian activity in the model without FOFR (Model without VIP/CNG coupling in ‘Modifications of the default parameter set’ section) within a period of several days. The $\nu_{\text{sPc}}$ parameter and parameters describing circadian regulation of firing rate were selected in such a way that the characteristic time of Per gene expression synchronization was approximately equal in all three models of circadian oscillations and at the same time, fast synchronization of firing rate was observed in Model with VIP/CNG coupling and with FOFR and Model with VIP/CNG coupling but without FOFR (see ‘Modifications of the default parameter set’ section for details)

1. **Chemical Species Used in the Model**

| *AC:* | Adenylate cyclase. |  |
| --- | --- | --- |
| *Gas.AC:* | Gs activated adenylate cyclase. |  |
| *GTP.Gas:* | Activated Gsalpha. |  |
| *cAMP:* | Cyclic AMP. |  |
| *Gas.AC_cyclase_complex:* | ATP bound Gs activated adenylate cyclase. |  |
| *R2C2:* | Protein Kinase A heterotetramer with two molecules of |  |
| *cAMP.R2C2:* | regulatory subunits and two molecules of catalytic subunits. |  |
|  | One molecule of cAMP bound PKA heterotetramer. |  |
| *cAMP2.R2C2:* | Two molecules of cAMP bound PKA heterotetramer. |  |
| *cAMP3.R2C2:* | Three molecules of cAMP bound PKA heterotetramer. |  |
| *cAMP4.R2C2:* | Four molecules of cAMP bound PKA heterotetramer. |  |
| *cAMP-PDE:* | cAMP phosphodiesterase. |  |
| *cAMP-PDE_PDE-complex:* | cAMP bound cAMP phosphodiesterase complex. |  |
| *cAMP-PDE*:* | Phosphorylated cAMP phosphodiesterase. |  |
| *cAMP-PDE*_PDE*_complex:* | cAMP bound phosphorylated cAMP phosphodiesterase |  |

complex.

*PKA-active_phosph_PDE_complex:* Activated PKA and cAMP phosphodiesterase complex.

| *PKA-active_c:* | Cytoplasmic activated PKA. |  |
| --- | --- | --- |
| *cAMP4.R2C:* | Four molecules of cAMP bound PKA with one catalytic |  |
| *cAMP4.R2:* | subunit released. |  |
|  | Four molecules of cAMP bound PKA with both catalytic |  |
| *Gbg:* | subunits released. |  |
|  | G-protein beta and gamma subunits. |  |
| *L.R.GDP.Gasbg:* | Ligand (VIP) bound receptor-G-protein complex. |  |
| *GDP.Gas:* | GDP bound Gsalpha. |  |
| *GDP.Gasbg:* | GDP bound Gs trimeric complex. |  |
| *R.GDP.Gasbg:* | Receptor-G-protein complex. |  |
| *Inhibited-PKA_c:* | Inhibitor bound cytoplasmic PKA. |  |
| *PKA-inhibitor_c:* | Cytoplasmic PKA inhibitor. |  |
| *R2:* | PKA regulatory unit dimer. |  |
| *R2C:* | PKA heterotetramer reforming intermediate. |  |
| *Inhibited-PKA_n:* | Inhibitor bound nuclear PKA. |  |
| *L.R:* | Ligand (VIP) bound *VPAC2* receptor. |  |
| *R:* | *VPAC2 r*eceptor. |  |
| *PKA-active_n:* | Nuclear active PKA. |  |
| *PKA-inhibitor_n:* | Nuclear PKA inhibitor. |  |
| *CREB:* | Inactive form transcription factor CREB. |  |
| *CREB.PKA-active_n:* | CREB and nuclear active PKA complex. |  |
| *CREB*:* | Phosphorylated active form CREB. |  |
| *CREB*_0_:* | Basal phosphorylated active form CREB. |  |
| *B_N_* | *Bmal1* mRNA |  |
| *Mp:* | *Per* mRNA. |  |
| *L.R_phosph* | *VIP-VPAC2* complex, phosphorylated by GRK |  |
| *L.R_phosph.GDP.Gasbg* | *VIP-VPAC2-G* complex, phosphorylated by GRK |  |
| *R_phosph* | *VPAC2*, phosphorylated by GRK |  |
| *R_phosph.GDP.Gasbg* | *VPAC2-G-protein* complex, phosphorylated by GRK |  |
| *GRK* | *GPCR* associated kinase |  |
| *GRK_active* | *GPCR* associated kinase, phoshorylated by *PKA* |  |
| *R_internal* | internalized *VPAC2* |  |
| *AC_inhibited* | Adenylatecyclase, inhibited by Gi |  |

1. **Model Equations**

Activation of adenylate cyclase by Gα subunit and its inhibition by Per gene product cascade, production of cAMP and its removing by phosphodiesterase (PDE) (Fig 1B, 1C):

= *k*_16_ [*Gas*.*AC*] *+k*_43_ [*Gas*.*AC*] − *k*_15_ [*AC*][*GTP*.*Gas*] *+k*_68_ [*AC_inhibited*]− *k*_67_ [*AC*][*M_p_*] (1)

= *k*_12_ [*Gas*.*AC* _ *cyclase* _ *complex*] − *k*_17_ [*cAMP*][*R*2*C*2]

*+ k*_18_ [*cAMP*.*R*2*C*2] − *k*_19_ [*cAMP*][*cAMP*.*R*2*C*2] + *k*_20_ [*cAMP*2.*R*2*C*2]

− *k*_21_ [*cAMP*][*cAMP*2.*R*2*C*2] + *k*_22_ [*cAMP*3.*R*2*C*2] − *k*_23_ [*cAMP*][*cAMP*3.*R*2*C*2]

*+ k*_24_ [*cAMP*4.*R*2*C*2] − *k*_35_ [*cAMP*][*cAMP* − *PDE*] + *k*_36_ [*cAMP* − *PDE* _ *PDE* _ *complex*]

− *k*_38_ [*cAMP*][*cAMP* − *PDE* *] + *k*_39_ [*cAMP* − *PDE* * _ *PDE* * _ *complex*] (2)

= *k*_31_ [*PKA* − *active* _ *phosph* − *PDE* _ *complex*]

− *k*_33_ [*cAMP* − *PDE*][*PKA* − *active* _ *c*] + *k*_34_ [*cAMP* − *PDE* *]

− *k*_35_ [*cAMP*][*cAMP* − *PDE*] + *k*_36_ [*cAMP* − *PDE* _ *PDE* _ *complex*]

+ *k*_37_ [*cAMP* − *PDE* _ *PDE* _ *complex*] (3)

$d$= *k*_32_ [ *PKA* − *active* _ *phosph* − *PDE* _ *complex*]

− *k*_34_ [*cAMP* − *PDE* *] − *k*_38_ [*cAMP*][*cAMP* − *PDE* *]

+ *k*_39_ [*cAMP* − *PDE* * _ *PDE* * _ *complex*]

+*k* _40_ [*cAMP* − *PDE* * _ *PDE* * _ *complex*] (4)

Activation of cytoplasmic PKA by cAMP and its inactivation by PKA-inhibitor (Fig. 1D):

= *k*_17_ [*cAMP*][*R*2*C*2] − *k*_18_ [*cAMP*.*R*2*C*2]

− *k*_19_ [*cAMP*][*cAMP*.*R*2*C*2] + *k*_20_ [*cAMP*2.*R*2*C*2] (5)

= *k*_19_ [*cAMP*][*cAMP*.*R*2*C*2] − *k*_20_ [*cAMP*2.*R*2*C*2]

− *k*_21_ [*cAMP*][*cAMP*2.*R*2*C*2] + *k*_22_ [*cAMP*3.*R*2*C*2] (6)

= *k*_21_ [*cAMP*][*cAMP*2.*R*2*C*2] − *k*_22_ [*cAMP*3.*R*2*C*2]

− *k*_23_ [*cAMP*][*cAMP*3.*R*2*C*2]+ *k*_24_ [*cAMP*4.*R*2*C*2] (7)

= *k*_28_ [*cAMP*4.*R*2*C*] − *k*_27_ [*cAMP*4.*R*2][*PKA* − *active* _ *c*]

− *k*_60_ [*cAMP*4.*R*2] (8)

= *k*_26_ [*cAMP*4.*R*2*C*2] − *k*_25_ [*cAMP*4.*R*2*C*][*PKA* − *active* _ *c*]

+ *k*_27_ [*cAMP*4.*R*2][*PKA* − *active* _ *c*] − *k*_28_ [*cAMP*4.*R*2*C*] (9)

= *k*_23_ [*cAMP*][*cAMP*3.*R*2*C*2] − *k*_24_ [*cAMP*4.*R*2*C*2] (10)

+ *k*_25_ [*cAMP*4.*R*2*C*2][*PKA* − *active* _ *c*] − *k*_26_ [*cAMP*4.*R*2*C*2]

Release of Gα and Gbg from VPAC2 receptors (Fig. 1B):

= *k*_10_ [*L*.*R*.*GDP*.*Gasbg*] − *k*_9_ [*Gbg*][*GDP*.*Gas*] + *k*_41_ [*GDP*.*Gasbg*] +

*k*_10_ [*L*.*R_phosph*.*GDP*.*Gasbg*] (11)

Activation of adenylate cyclase by Gα (Fig 1B):

= *k*_11_ [*GTP*.*Gas*] − *k*_9_ [*Gbg*][*GDP*.*Gas*] + *k*_43_ [*Gas*.*AC*] (12)

= *k*_9_ [*Gbg*][*GDP*.*Gas*] − *k*_3_ [*GDP*.*Gasbg*][*L*.*R*] − *k*_65_ [*GDP*.*Gasbg*][*L*.*R*]

-*k*_66_ [*GDP*.*Gasbg*][*R_phosph*] + *k*_6_ [*R_phosph*.*GDP*.*Gasbg*] + *k*_4_ [*L*.*R_phosph*.*GDP*.*Gasbg*]

+ *k*_4_ [*L*.*R*.*GDP*.*Gasbg*] − *k*_5_ [*GDP*.*Gasbg*][*R*] + *k*_6_ [*R*.*GDP*.*Gasbg*] − *k*_41_ [*GDP*.*Gasbg*] (13)

= *k*_15_ [*AC*][*GTP*.*Gas*] − *k*_16_ [*Gas*.*AC*] − *k*_13_ [*Gas*.*AC*][*ATP*]

+ *k*_12_ [*Gas*.*AC* _ *cyclase* _ *complex*] + *k*_14_ [*Gas*.*AC* _ *cyclase* _ *complex*] − *k*_43_ [*Gas*.*AC*] (14)

= *k*_10_ [*L*.*R*.*GDP*.*Gasbg*] + *k*_10_ [*L*.*R_phosph*.*GDP*.*Gasbg*] − *k*_11_ [*GTP*.*Gas*] − *k*_15_ [*AC*][*GTP*.*Gas*]

+ *k*_16_ [*Gas*.*AC*]+ *k*_41_ [*GDP*.*Gasbg*] (15)

Inhibition of PKA by PKA-inhibitor in the cytoplasm (Fig. 1D):

= *k*_29_ [*PKA* − *active* _ *c*][*PKA* −*inhibitor* _ *c*]

− *k*_30_ [*inhibited* − *PKA* _ *c*] − *k*_53_ [*inhibited* − *PKA* _ *c*][*R*2]

+ *k*_54_ [*PKA* − *inhibitor* _ *c*][*R*2*C*] − *k*_55_ [*inhibited* − *PKA* _ *c*][*R*2*C*]

+ *k*_56_ [*PKA* − *inhibitor* _ *c*][*R*2*C*2] + *k*_51_ [*inhibited* − *PKA* _ *n*]

− *k*_52_ [*inhibited* − *PKA* _ *c*] (16)

Activation of VPAC2 receptors by VIP and release of Gα and Gbg (Fig. 1F, 1B):

= *k*_1_ [*R*][*L*]− *k*_2_ [*L*.*R*] − *k*_3_ [*GDP*.*Gasbg*][*L*.*R*] + *k*_4_ [*L*.*R*.*GDP*.*Gasbg*]

+ *k*_10_ [*L*.*R*.*GDP*.*Gasbg*] − *k*_63_ [*L*.*R*][*GRK_active*] + *k*_64_ [*L*.*R_phosph*] - *k*_71_ [*GRK_active*][*L.R*] (17)

= *k*_3_ [*GDP*.*Gasbg*][*L*.*R*] − *k*_4_ [*L*.*R*.*GDP*.*Gasbg*]

+ *k*_7_ [*R*.*GDP*.*Gasbg*][*L*] − *k*_8_ [*L*.*R*.*GDP*.*Gasbg*] − *k*_10_ [*L*.*R*.*GDP*.*Gasbg*]

− *k*_63_ [*L*.*R*.*GDP*.*Gasbg*][*GRK_active*] + *k*_64_ [*L*.*R_phosph*.*GDP*.*Gasbg*] (18)

Activation of cytoplasmic PKA by cAMP and its inactivation by PKA-inhibitor (Fig 1D):

= *k*_26_ [*cAMP*4.*R*2*C*2] − *k*_25_ [*cAMP*4.*R*2*C*][*PKA* − *active* _ *c*]

+ *k*_28_ [*cAMP*4.*R*2*C*] − *k*_27_ [*cAMP*4.*R*2][*PKA* − *active* _ *c*]

− *k*_29_ [*PKA* − *active* _ *c*] [*PKA* − *inhibitor* _ *c*] + *k*_30_ [*inhibited* − *PKA* _ *c*]

− *k*_33_ [*cAMP* − *PDE*][*PKA* − *active* _ *c*] + *k*_31_ [*PKA* − *active* _ *phosph* − *PDE* _ *complex*]

+ *k*_32_ [*PKA* − *active* _ *phosph* − *PDE* _ *complex*] − *k*_44_ [*PKA* − *active* _ *c*]

+ *k*_45_ [*PKA* − *active* _ *n*] (19)

= *k*_30_ [ *inhibited PKA* _ *c*]

− *k*_29_ [*PKA active* _ *c*][*PKA* − *inhibitor* _ *c*] − *k*_57_ [*PKA* − *inhibitor* _ *c*]

+ *k*_58_ [*PKA* − *inhibitor* _ *n*] *+ k*_55_ [*inhibited* − *PKA* _ *c*][*R*2*C*]

− *k*_56_ [*PKA* − *inhibitor* _ *c*][*R*2*C*2] + *k*_53_ [*inhibited* − *PKA* _ *c*][*R*2]

− *k*_54_ [*PKA* −*inhibitor* _ *c*][*R*2*C*] (20)

Activation of VPAC2 receptors by VIP and release of Gα and Gbg (Fig 1F, 1B):

= *k*_2_ [*L*.*R*] − *k*_1_ [*R*][*L*] − *k*_5_ [*GDP*.*Gasbg*][*R*] + *k*_6_ [*R*.*GDP*.*Gasbg*]

− *k*_63_ [*GRK_active*][*R*] + *k*_64_ [*R_phosph*] + *k*_70_ [*R_internal*] (21)

= *k*_5_ [*GDP*.*Gasbg*][*R*] − *k*_6_ [*R*.*GDP*.*Gasbg*] −

*k*_7_ [*R*.*GDP*.*Gasbg*][*L*] + *k*_8_ [*L*.*R*.*GDP*.*Gasbg*]

− *k*_63_ [*GRK_active*][*R. GDP*.*Gasbg*] + *k*_64_ [*R_phosph. GDP*.*Gasbg*] (22)

Activation of cytoplasmic PKA by cAMP and its inactivation by PKA-inhibitor (Fig 1D):

= *k*_18_ [*cAMP*.*R*2*C*2] − *k*_17_ [*cAMP*][*R*2*C*2] + *k*_55_ [*inhibited* − *PKA* _ *c*][*R*2*C*]

− *k*_56_ [*PKA* − *inhibitor* _ *c*][*R*2*C*2] (23)

Activation of adenylate cyclase (AC) by Gα subunit and its inhibition by Per product cascade, production of cAMP and its removing by PDE (Fig 1B, 1C):

= *k*_13_ [*Gas*.*AC*] [*ATP*]

− *k*_12_ [*Gas*.*AC* _ *cyclase* _ *complex*] − *k*_14_ [*Gas*.*AC* _ *cyclase* _ *complex*] (24)

= *k*_35_ [*cAMP*][ *cAMP - PDE*]

− *k*_36_ [*cAMP* − *PDE* _ *PDE* _ *complex*] − *k*_37_ [*cAMP* − *PDE* _ *PDE* _ *complex*] (25)

= *k*_38_ [*cAMP*] [*cAMP -PDE* *]

− *k*_39_ [*cAMP* − *PDE* * _ *PDE* * _ *complex*]

− *k*_40_ [*cAMP* − *PDE* * _ *PDE* * _ *complex*] (26)

= *k*_33_ [*cAMP -PDE*][*PKA* − *active* _ *c*]

− *k*_31_ [*PKA* − *active* _ *phosph* − *PDE* _ *complex*]

− *k*_32_ [*PKA* − *active* _ *phosph* − *PDE* _ *complex*] (27)

Diffusion of PKA to the nucleus and activation of CREB (Fig. 1E):

= *k*_44_ [*PKA* − *active* _ *c*] − *k*_45_ [*PKA* − *active* _n]

− *k*_46_ [*CREB*][*PKA* − *active* _ *n*] + *k*_47_ [*CREB*.*PKA* − *active* _ *n*]

+ *k*_48_ [*CREB*.*PKA* − *active* _ *n*] − *k*_49_ [*PKA* − *active* _ *n*][*PKA* − *inhibitor* _ *n*]

+ *k*_50_ [*inhibited* − *PKA* _ *n*] (28)

= *k*_49_ [*PKA* − *active* _ *n*][*PKA* −*inhibitor* _ *n*]

− *k*_50_ [*inhibited* − *PKA* _ *n*] − *k*_51_ [*inhibited* − *PKA* _ *n*] + *k*_52_ [*inhibited* − *PKA* _ *c*] (29)

= *k*_57_ [*PKA* − *inhibitor* _ *c*] − *k*_58_ [*PKA* − *inhibitor* _ *n*]

− *k*_49_ [*PKA* − *active* _ *n*][*PKA* − *inhibitor* _ *n*] + *k*_50_ [*inhibited* − *PKA* _ *n*] (30)

= *k*_47_ [*CREB*.*PKA* − *active* _ *n*] − *k*_46_ [*CREB*][*PKA* − *active* _ *n*]

+ *k*_59_ [*CREB* *] (31)

= *k*_48_ [*CREB*.*PKA* − *active* _ *n*] − *k*_59_[*CREB* *] (32)

= *k*_46_ [*CREB*][*PKA* − *active* _ *n*]

−[*CREB*.*PKA* − *active* _ *n*] − *k*_48_ [*CREB*.*PKA* − *active* _ *n*] (33)

Activation of PKA by cAMP and its inactivation by PKA-inhibitor in cytoplasm (Fig. 1D):

= *k*_60_ [*cAMP*4.*R*2] − *k*_53_ [*inhibited* − *PKA* _ *c*][*R*2] + *k*_54_ [*PKA* − *inhibitor* _ *c*][*R*2*C*] (34)

= *k*_53_ [*inhibited* − *PKA* _ *c*][*R*2] − *k*_54_ [*PKA* − *inhibitor* _ *c*][*R*2*C*]

− *k*_55_ [*inhibited* − *PKA* _ *c*][*R*2*C*]+ *k*_56_ [*PKA* − *inhibitor* _ *c*][*R*2*C*2] (35)

Activation of Per gene expression by CREB (Fig. 1E):

= (36)

Phosphorylation of VPAC2 receptors by GRK and activation of phosphorylated receptors by VIP (Fig. 1F):

= *k*_1_ [*R_phosph*][*L*] − *k*_2_ [*L*.*R*]

− *k*_65_ [*GDP*.*Gasbg*][*L*.*R*] + *k*_4_ [*L*.*R*.*GDP*.*Gasbg*]

*+ k*_10_ [*L*.*R_phosph*.*GDP*.*Gasbg*]− *k*_42_ [*L*.*R_phosph*]

+ *k*_63_ [*L*.*R_phosph*][*GRK_active*] − *k*_64_ [*L*.*R_phosph*] (37)

= *k*_65_ [*GDP*.*Gasbg*][*L*.*R_phosph*]

− *k*_4_ [*L*.*R_phosph*.*GDP*.*Gasbg*] + *k*_7_ [*R_phosph*.*GDP*.*Gasbg*][*L*]

− *k*_8_ [*L*.*R_phosph*.*GDP*.*Gasbg*] − *k*_10_ [*L*.*R_phosph*.*GDP*.*Gasbg*] (38)

= *k*_2_ [*L*.*R_phosph*] − *k*_1_ [*R_phosph*][*L*] − *k*_66_ [*GDP*.*Gasbg*][*R_phosph*]+

+ *k*_6_ [*R_phosph*.*GDP*.*Gasbg*] + *k*_63_ [*GRK_active*][*R*] −*k*_64_ [*R_phosph*] − *k*_42_ [*R_phosph*] (39)

= *k*_66_ [*GDP*.*Gasbg*][*R_phosph*] − *k*_6_ [*R_phosph*.*GDP*.*Gasbg*]

−*k*_7_ [*R_phosph*.*GDP*.*Gasbg*][*L*] + *k*_8_ [*L*.*R_phosph*.*GDP*.*Gasbg*] (40)

Activation of GRK by PKA (Fig. 1F):

= *k*_62_ [*GRK_active*] − *k*_61_ [*GRK*]([ *PKA* − *active* _c])^G^ (41)

=− *k*_62_ [*GRK_active*] + *k*_61_ [*GRK*]([ *PKA* − *active* _c])^G^ (42)

Inhibition of adenylat cyclase (AC) by Per gene product cascade (Fig. 1B):

= − *k*_68_ [*AC_inhibited*] + *k*_67_ [*AC*][*M_p_*] (43)

Release and diffusion of VIP (single cell):

= (44)

Internalization of phosphorylated VPAC_2_ receptors (Fig. 1D):

= *k*_42_ [*L*.*R_phosph*] + *k*_71_ [*GRK_active*][*L.R*]

+*k*_42_ [*R_phosph*] − *k*_70_ [*R_internal*] (45)

The firing rate of a neuron $F$ was described by the equation:

(46)

where function $\left| x \right|_{+}=x$ for $x\geq0$ and $\left| x \right|_{+}=0$ for $x<0$

$I_{\text{CNG}}$ is a current through CNG channels:

(47)

For the model of network of interacting SCN neurons (see ‘A model for a heterogeneous cell population’ in the main text) Eq. 44 was modified for each cell $i$ in the network of $N_{\text{cells}}$ neurons to introduce the coupling of oscillators:

= (48)

When circadian rhythm synchronization was modeled (see ‘Modeling the circadian synchronization in the SCN network with FOFR’ in the main text), two groups of CNG channels were introduced. One was coupled with VPAC2 receptors:

(49)

(50)

and the other group was activated by other local cAMP sources whose activity was not controlled by cytosolic signaling from VPAC_2_ receptors (), but conductivity of these CNG channels was modulated by the Per gene expression:

(51)

(52)

1. **Table S1. Parameter values**

| Parameter | Units | Value | Description |  |
| --- | --- | --- | --- | --- |
|  |  |  |  |  |
| *k*_1_ | nM^-1^.s^-1^ | 0.0001 | k_f_ for ligand binding to standalone receptors. |  |
|  |  |  |  |  |
|  |  |  |  |  |
| *k_2_* | _s_-1 | 0.1 | k_b_ for ligand binding to standalone receptors. |  |
| *k_3_* | nM^-1^.s^-1^ | 0.001 | k_f_ for G-protein coupling to ligand-receptor |  |
|  |  |  | complex. |  |
|  |  |  |  |  |
| *k_4_* | _s_-1 | 0.3 | k_b_ for G-protein coupling to ligand-receptor |  |
|  |  |  | complex |  |
|  |  |  |  |  |
| *k_5_* | nM^-1^.s^-1^ | 0.0002 | k_f_ for G-protein coupling to receptor. |  |
| *k_6_* | _s_-1 | 0.1 | k_b_ for G-protein coupling to receptor. |  |
| *k_7_* | nM^-1^.s^-1^ | 0.01 | k_f_ for ligand binding to receptor G-protein complex |  |
| *k_8_* | _s_-1 | 0.1 | k_b_ for ligand binding to receptor G-protein complex |  |
| *k_9_* | nM^-1^.s^-1^ | 0.006 | k_f_ for G-protein subunit association. |  |
| *k10* | _s_-1 | 0.025 | k_f_ for G-protein activation |  |
| *k11* | _s_-1 | 0.066667 | k_f_ for intrinsic GTP hydrolysis. |  |
| *k12* | _s_-1 | 0.00214275 | k_3_ for cAMP production by activated AC |  |
|  |  |  |  |  |
|  |  |  |  |  |
| *k13* | nM^-1^.s^-1^ | 0.0045 | k_1_ for ATP binding to activated AC. |  |
| *k14* | _s_-1 | 90 | k_2_ for ATP and AC dissociation. |  |
| *k15* | nM^-1^.s^-1^ | 0.25 | k_f_ for activated G protein binding to AC. |  |
| *k16* | _s_-1 | 1 | k_b_ for activated G-protein binding to AC. |  |
| *k17* | nM^-1^.s^-1^ | 0.027 | k_f_ for cAMP binding to PKA regulatory site B1. |  |
| *k18* | _s_-1 | 33 | k_b_ for cAMP binding to PKA regulatory site B1. |  |
| *k19* | nM^-1^.s^-1^ | 0.108 | k_f_ for cAMP binding to PKA regulatory site B2. |  |
| *k20* | _s_-1 | 0.66 | k_b_ for cAMP binding to PKA regulatory site B2. |  |
| *k21* | nM^-1^.s^-1^ | 0.108 | k_f_ for cAMP binding to PKA regulatory site A1. |  |
| *k22* | _s_-1 | 0.66 | k_b_ for cAMP binding to PKA regulatory site A1. |  |
| *k23* | nM^-1^.s^-1^ | 0.216 | k_f_ for cAMP binding to PKA regulatory site A2. |  |
| *k24* | _s_-1 | 0.325 | k_b_ for cAMP binding to PKA regulatory site A2. |  |
| *k25* | nM^-1^.s^-1^ | 0.108 | k_b_ for releasing PKA catalytic unit C1. |  |
| *k26* | _s_-1 | 1.40625 | k_f_ for releasing PKA catalytic unit C1. |  |
| *k27* | nM^-1^.s^-1^ | 0.108 | k_b_ for releasing PKA catalytic unit C2. |  |
| *k28* | _s_-1 | 1.40625 | k_f_ for releasing PKA catalytic unit C2. |  |
| *k29* | nM^-1^.s^-1^ | 0.060 | k_f_ for cytoplasmic inhibitor binding to active PKA. |  |
| *k30* | _s_-1 | 0.1 | k_b_ for cytoplasmic inhibitor binding to active PKA. |  |
| *k31* | _s_-1 | 3.6 | k_2_ for PKA phosphorylating PDE. |  |
| *k32* | _s_-1 | 0.9 | k_3_ for PKA phosphorylating PDE. |  |
| *k33* | nM^-1^.s^-1^ | 0.0029 | k_1_ for PKA phosphorylating PDE. |  |
| *k34* | _s_-1 | 0.4 | rate for phopho-PDE dephosphorylation. |  |
| *k35* | nM^-1^.s^-1^ | 0.005040068544932 | k_1_ for basal PDE converting cAMP to AMP. |  |
| *k36* | _s_-1 | 40 | k_2_ for basal PDE converting cAMP to AMP. |  |
| *k37* | _s_-1 | 0.066125 | k_3_ for basal PDE converting cAMP to AMP. |  |
| *k38* | nM^-1^.s^-1^ | 0.005040068544932 | k_1_ for active PDE converting cAMP to AMP. |  |
| *k39* | _s_-1 | 50 | k_2_ for active PDE converting cAMP to AMP. |  |
| *k40* | _s_-1 | 50 | k_3_ for active PDE converting cAMP to AMP. |  |
| *k41* | _s_-1 | 0.000035 | rate for basal G-protein activation. |  |
| ***k42*** | **_s_-1** | **0.0033333** | **rate for phosphorylated-receptor internalization.** |  |
| *k43* | _s_-1 | 30 | rate for GTP hydrolysis. |  |
| *k44* | _s_-1 | 0.0025 | k_f_ for active PKA nuclear translocation. |  |
| *k45* | _s_-1 | 0.00002 | k_b_ for active PKA nuclear translocation. |  |
| *k46* | nM^-1^.s^-1^ | 0.45 | k_1_ for PKA phosphorylating CREB. |  |
| *k47* | _s_-1 | 0.0225 | k_2_ for PKA phosphorylating CREB. |  |
| *k48* | _s_-1 | 450 | k_3_ for PKA phosphorylating CREB. |  |
| *k49* | nM^-1^.s^-1^ | 0.030 | k_f_ for nuclear inhibitor binding to nuclear PKA. |  |
| *k50* | _s_-1 | 0.2 | k_b_ for nuclear inhibitor binding to nuclear PKA. |  |
| *k51* | _s_-1 | 0.0005 | k_f_ for inhibited PKA translocation to cytoplasm. |  |
| *k52* | _s_-1 | 0.005 | k_b_ for inhibited PKA translocation to cytoplasm. |  |
| *k53* | nM^-1^.s^-1^ | 0.15 | k_f_ for first PKA binding to regulatory dimer. |  |
| *k54* | nM^-1^.s^-1^ | 0.0000004523 | k_b_ for first PKA binding to regulatory dimer. |  |
| *k55* | nM^-1^.s^-1^ | 0.15 | k_f_ for second PKA binding to regulatory dimer. |  |
| *k56* | nM^-1^.s^-1^ | 0.0000000175 | k_b_ for second PKA binding to regulatory dimer. |  |
| *k57* | _s_-1 | 0.15 | k_f_ for PKA inhibitor nuclear translocation. |  |
| *k58* | _s_-1 | 0.005 | k_b_ for PKA inhibitor nuclear translocation. |  |
| *k59* | _s_-1 | 0.05 | CREB_P_ dephosphorylation rate. |  |
| *k60* | _s_-1 | 0.0086 | rate for PKA Regulatory unit and cAMP dissociate. |  |
| ***k61*** | **nM^-4^.s^-1^** | **300** | **Activation of GRK by PKA_active** |  |
| ***k62*** | **_s_-1** | **0.02** | **Deactivation of GRK** |  |
| ***k63*** | **nM^-1^.s^-1^** | **0.0166667** | **Phosphorylation of VPAC2 by GRK** |  |
| ***k64*** | **_s_-1** | **0.00066667** | **Dephosphorylation of VPAC2** |  |
| ***k65*** | **nM^-1^.s^-1^** | **0.0001** | **Binding of VIP_VPAC_phosph. with Gs** |  |
| ***k66*** | **nM^-1^.s^-1^** | **0.00002** | **Binding of VPAC_ phosph. with Gs** |  |
| *k67* | nM^-1^.s^-1^ | 0 | Inactivation of AC by Gi |  |
| *k68* | _s_-1 | 0.01 | Reactivation of AC |  |
| *k69* | nM^-1^.s^-1^ | 0 | Inactivation of AC by G_i_ dependent on Per |  |
| ***k70*** | **_s_-1** | **0.00066667** | **Insertion of new VPAC2 receptors** |  |
| ***k71*** | **nM^-1^.s^-1^** | **0 or 0.0033333 ^**^** | **Internalization of receptors without phosphorylation** |  |
| *ν_sP_* | nMs^-1^ | 0.00041667 | Maximum rate for Per mRNA synthesis. |  |
| *KAP* |  |  | Activation constant for enhancement of Per gene |  |
|  | nM | 0.7 | expression by Bmal1/Clk |  |
| *ν_sPc_* | nMs^-1^ | -0.3 | Maximum enchancement of the rate for Per mRNA synthesis by CREB |  |
|  |  |  |  |  |
|  | nM | 1.3 | CREB concentration for half-activation of Per mRNA synthesis enchancement |  |
| ***KAPc*** | **nM** | **0.7** | **Activation constant for enhancement of Per**  **expression by CREB_P_** |  |
| *k_mp_* | nM | 0.31 | Michaelis constant for degradation of Per mRNA |  |
|  |  |  |  |  |
| *v_mp_* | nMs^-1^ | 0.00061112 | Maximum rate for Per mRNA degradation. |  |
|  |  |  |  |  |
| *k_dmp_* | _s_-1 | 0.00000277778 | Nonspecific degradation rate constant for mRNA |  |
|  |  |  |  |  |
| ***V*** |  | **3** | **Cooperativity of CNG channels activation** |  |
| ***G*** |  | **3** | **Cooperativity of GRK activation by PKA** |  |
| ***D*** |  | **0.2-10** | **Coefficient that regulate VIP exchange** |  |
| ***Θ*** | **_s_-1** | **0.08** | **Threshold for AP generation** |  |
|  | **nM** | **25** | **VIP packet released after a single AP** |  |
|  | **s** | **2** | **Decay of VIP through diffusion** |  |
|  | **_s_-1** | **0.25** | **Maximal conductivity of CNG channels** |  |
|  |  | **0.2** | **Part of CNG channels regulated by Per** |  |
|  | **nM** | **0.315** | **Concenentration of cAMP near *Per* regulated CNG channels** |  |
|  | **nM** | **0.50396842** | **cAMP concentration of half-activation of CNG channels** |  |
| ***L_0_*** | **nM** | **1.5** | **Steady state VIP concentration** |  |
| nc |  | 2 | Degree of cooperativity of CREB interaction with *Per* gene expression |  |
| n |  | 2 | Degree of cooperativity of transcription factors |  |
|  |  |  |  |  |

^**^  - first value is default, 2^nd^ – was used when internalization of VPAC_2_ receptors without their desensitization was modeled (see 3 in the ‘Basic assumptions of the model’ section of Materials and Methods)

1. **Table 2. Initial Concentrations**

| Molecule | Concentration (nM) |
| --- | --- |
|  |  |
| AC | 12.965 |
|  |  |
| cAMP | 0.14625 |
|  |  |
| cAMP-PDE | 500.08 |
|  |  |
| cAMP-PDE* | 0.00040043 |
|  |  |
| cAMP.R2C2 | 0.059672 |
|  |  |
| cAMP2.R2C2 | 0.0014268 |
|  |  |
| cAMP3.R2C2 | 0.000032869 |
|  |  |
| cAMP4.R2 | 0.00098071 |
|  |  |
| cAMP4.R2C | 0.00000060392 |
|  |  |
| cAMP4.R2C2 | 0.00000059979 |
|  |  |
| Gbg | 0.012996 |
|  |  |
| GDP.Gas | 448.82 |
|  |  |
| GDP.Gasbg | 999.91 |
|  |  |
| Gas.AC | 0.0011423 |
|  |  |
| GTP.Gas | 0.010925 |
|  |  |
| inhibited-PKA | 0.0026027 |
|  |  |
| L.R | 0 |
|  |  |
| L.R.GDP.Gasbg | 0 |
|  |  |
| PKA-active_c | 0.00058223 |
|  |  |
| PKA-inhibitor_c | 7.8649 |
|  |  |
| R | 27.806 |
|  |  |
| R.GDP.Gasbg | 55.607 |
|  |  |
| R2C2 | 498.67 |
|  |  |
| Gas.AC_cyclase_complex | 0.28556 |
|  |  |
| cAMP-PDE_PDE_complex | 0.0092003 |
|  |  |
| cAMP-PDE*_PDE*_complex | 0.0000000029214 |
|  |  |
| PKA-active_phosph-PDE_complex | 0.00017614 |
|  |  |
| AMP | 1000000 |
|  |  |
| ATP | 5000000 |
|  |  |
| L | 0 |
|  |  |
| L.R_phosph | 0 |
| L.R_phosph.GDP.Gasbg | 0 |
| R_phosph | 0 |
| R_internal | 0 |
| R_phosph.GDP.Gasbg | 0 |
| GRK | 2900 |
| GRK_active | 0 |
| AC_inhibited | 0 |
| PKA_active_n | 0.00080531 |
|  |  |
| inhibitied-PKA_n | 0.028495 |
|  |  |
| PKA-inhibitor_n | 235.95 |
|  |  |
| CREB | 79.425 |
|  |  |
| CREB* | 0.57042 |
|  |  |
| CREB.PKA_active_n | 0.000063959 |
|  |  |
| R2 | 0.0038906 |
|  |  |
| R2C | 0.17982 |
|  |  |
| *Bmal1* mRNA | 0 |
| *Per* mRNA | 0 |
|  |  |

1. **Modifications of the default parameter set**

For the model of the circadian activity synchronization in the SCN network with FOFR the default parameter set was modified (see ’Modeling the circadian synchronization in the SCN network with FOFR’ in the Materials and Methods section of the main text).We considered three models of circadian regulation of neuron firing:

1) Model without VIP/CNG coupling: VPAC2 receptors are not directly coupled to CNG channels and firing rate is regulated via inhibition of CNG channels by Per gene product.

=1 (Eq. 50, 51); *=0.6* (Eq. 51)*; Θ=0.1644* (Eq. 46)*; =-0.45* (Eq. 36)*;*

2) Model with VIP/CNG coupling with FOFR: There are two pools of CNG channels: most (80%) of CNG channels are coupled to VPAC_2_ receptors and do not depend on circadian clock molecular signals, and the minority of CNG channels are regulated by Per gene expression, as in the 1^st^ model.

=0.2; *=0.315;* *=-4.5; Θ= 0.1349; =12* (Eq. 48);

3) Model with VIP/CNG coupling without FOFR: There are two pools of CNG channels as in the Model with VIP/CNG coupling with FOFR, but the desensitization of VPAC_2_ receptors was switched off by setting initial GRK level to zero and parameters of the model were selected such that the system was close to the transition from monostability to bistability.

=0.1; *=0.515;* *=-0.3; Θ=0.62423; =1.7;* =2.9 nM (Eq. 36);

[GRK]=0 nM;

The parameter set for the Model with VIP/CNG coupling without FOFR was selected on the basis of the analysis of firing rate synchronization in the network without FOFR. The main reason for the improvement of the synchronization of firing rate oscillations with respect to the oscillations of Per gene expression in the network with fast cytosolic signaling from VPAC_2_ to cAMP-dependent ion channels (CNG channels in the model) is efficient amplification and averaging of VIP signal in the VPAC_2_ – cAMP – VIP positive feedback loop. Indeed, let suppose that amplitude of circadian oscillations of the membrane current component ($I_{\text{CNG},2}$) through the group of channels directly modulated by Per gene expression is , and that standard deviation of the phases of these oscillations over the population of neurons in the network is ${\Delta\varphi}_{2}$. In the case of intensive VIP exchange between the neurons, the current through the group of channels modulated by VIP concentration via cytosolic signaling from VPAC_2_ receptors to CNG channels ($I_{\text{CNG},1}$) should oscillate approximately in synchrony () in different cells of the network since all cells experience approximately the same oscillating VIP concentration. As result, if ${\Delta\varphi}_{2}$is sufficiently small then the standard deviation of the phases of circadian oscillations of the total current through CNG channels () in the population of neurons can be approximated as ,

where is the average amplitude of the circadian oscillations of $I_{\text{CNG},1}$. The best conditions for synchronization of firing rate oscillations (the amplitude of which is proportional to the ) could be obtained from the analysis of the model behavior in the $I_{\text{CNG},1}$ - VIP phase plane (Figure A1). It can be seen from this figure that when $I_{\text{CNG},2}$ changes on , the VIP- nullcline (red line) shifts down (also on ). As result of this shift the equilibrium value of $I_{\text{CNG},1}$(which could be obtained as intersection point of nullclines) also shifts and the amplitude of this shift in the linear approximation could be estimated as:

,

where $\alpha$and $\beta$ are angles of slope of VIP-$I_{\text{CNG},1}$ and $I_{\text{CNG},1}$-VIP nullclines near the intersection point, respectively. Thus, the best conditions for synchronization of firing rate oscillations (the amplitude of which linearly depends on ) are conditions when |$\alpha$-$\beta$| are small, i.e. when the system is close to the transition from monostability ($\alpha$< $\beta$) to bistability ($\alpha$> $\beta$).


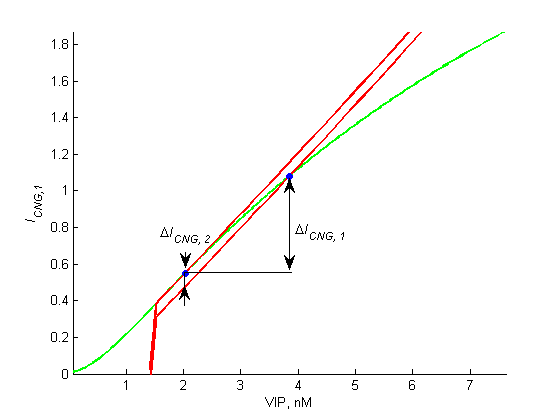


**Figure A1. Efficient amplification and averaging of VIP signal in VPAC_2_ – cAMP – VIP positive feedback loop.**

VIP and $I_{\text{CNG},1}$ nullclines for the FOFR model with parameters close to the parameter set for the Model with VIP/CNG coupling but without FOFR. $I_{\text{CNG},1}$ nullclines before and after alteration of the CNG channels current component directly modulated by Per expression ($I_{\text{CNG},2}$) are shown in red. VIP nullcline is shown in green. When $I_{\text{CNG},2}$ changes on the equilibrium value of $I_{\text{CNG},1}$ current, which is given by the nullclines intersection point, shifts on . The ratio of to will be maximal when the slopes of VIP and $I_{\text{CNG},1}$ nullclines are close to each other.

1. **Sensitivity of synchronization of the circadian oscillations of firing rate to the model parameters**

The extent to which synchronization of the circadian oscillations of firing rate can be improved with respect to the synchronization of Per gene expression when fast cytosolic VPAC_2_ – cAMP – VIP positive feedback loop was added to the system depends on the existence of FOFR. Even if it is possible to obtain such synchronization improvement in the model without FOFR (for example, it is observed in the Model with VIP/CNG coupling without FOFR), this improvement is restricted to the relatively narrow range of the model parameters. To demonstrate this, we have repeated experiment with circadian oscillations synchronization (1^st^ experiment in the last section of Results) with systematically changed parameters ${\Delta L}_{\text{AP}}$ and $\alpha_{\text{id}}$. Evolution of the network activity started from the same initial distribution of circadian oscillations phases. The standard deviation of circadian oscillations of firing rate phases in the network was measured within the first day after the coupling of oscillators was switched on. The dependences of this standard deviation (in % of SD for default model) on the magnitude of parameter change (in %) are shown in **Table S3.** It can be seen that when FOFR in the network was absent (Model with VIP/CNG coupling without FOFR) the improvement of the synchronization of firing rate circadian oscillations was several times more sensitive to the model parameters compared to the case when FOFR was observed (Model with VIP/CNG coupling with FOFR).

**Table S3. Sensitivity of the firing rate circadian oscillations synchronization to the model parameters perturbations**

|  |  |  |  |  |  |
| --- | --- | --- | --- | --- | --- |
| Parameter change | 0%_ | +20% | +15% | +10% | -10% |
| Model with VIP/CNG coupling with FOFR | 100 | 87 | 79 | 75 | 157 |
| Model with VIP/CNG coupling without FOFR | 100 | 243 | 178 | 104 | 396 |
|  |  |  |  |  |  |
| Parameter change | 0% | +20% | +15% | +10% | -5% |
| Model with VIP/CNG coupling with FOFR | 100 | 83 | 88 | 88 | 94 |
| Model with VIP/CNG coupling without FOFR | 100 | 768 | 365 | 233 | 105 |
