# Supplementary material for: A Model for the Fast Synchronous Oscillations of Firing Rate in Rat Suprachiasmatic Nucleus Neurons Cultured in a Multielectrode Array Dish
Source: PLoS One. 2014 Sep 5;9(9):e106152. doi: 10.1371/journal.pone.0106152 (PMC4156468; doi:10.1371/journal.pone.0106152)
Supplement: Figure S1 — Fast oscillations of firing rate (FOFR) recorded in dispersed rat SCN neurons in multi-electrode array dishes (MEDs). (DOC) [file pone.0106152.s002.doc]

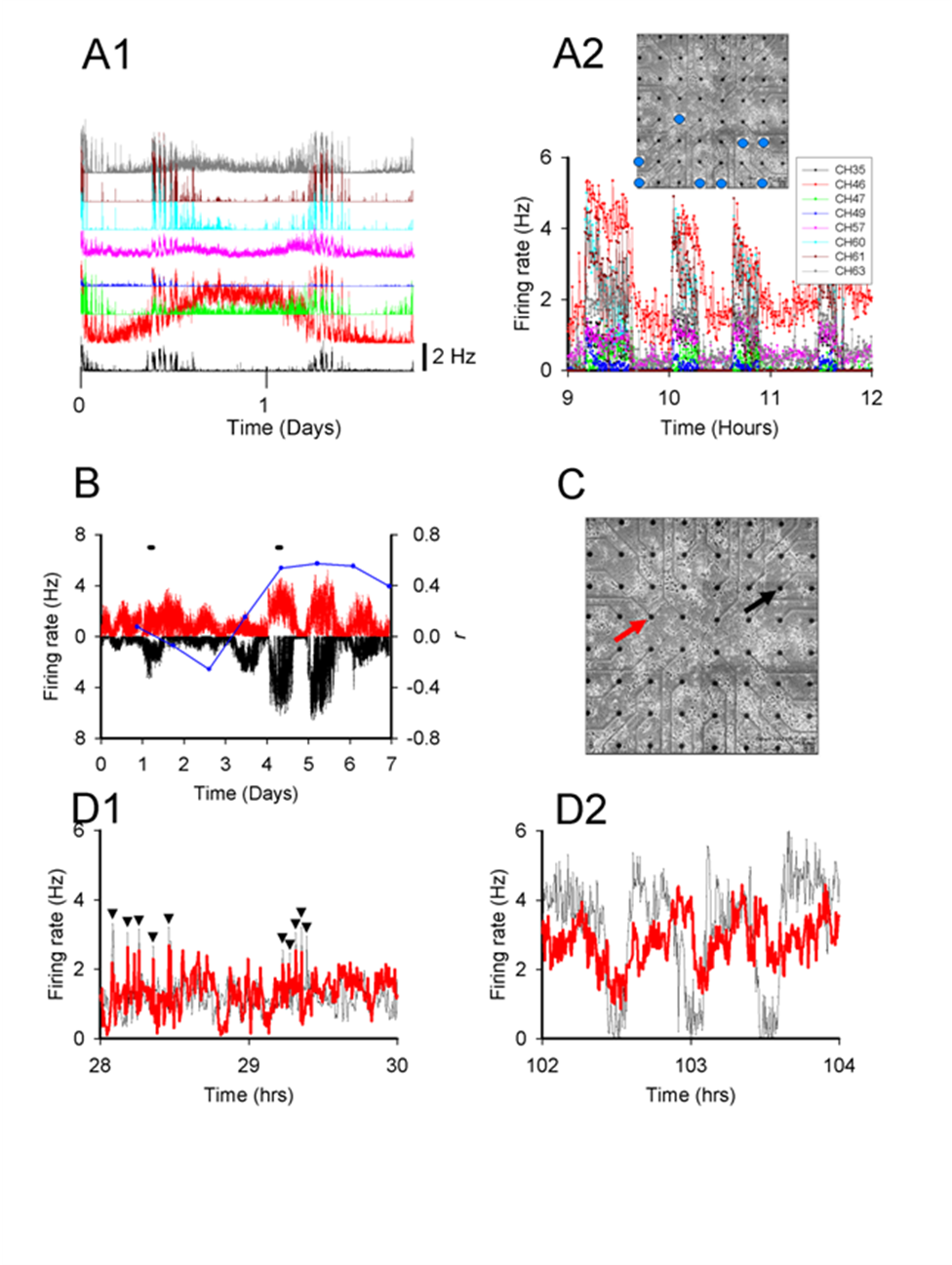


Fig. S1. Fast oscillations of firing rate (FOFR) recorded in dispersed rat SCN neurons in multi-electrode array dishes (MEDs)

A1, A2 Neural network activity showing both circadian cycles and FOFR. A1. Firing rate of eight neurons, positions of which on MED are shown in upper right corner in (A2). A2. Plot of firing rates of the same neurons as in (A1) at expanded time scale shows details of FOFR patterns.

B. Transition from non-circadian to circadian firing pattern coincides with increasing RtR correlation for two neurons shown in (C). Firing rate of neurons (#23, black; #27,

red) over seven days. For better visibility, firing rate of one neuron is directed downward. Blue plot (right scale) shows time course of RtR correlation with 1-day bin. Thick

black horizontal bars near top show intervals presented in (D1 and D2) at expanded time scale.

C. Positions of two neurons on MED for which transition from non-circadian to circadian firing pattern was observed. D1. Firing rates of neurons during the period of non-circadian

and uncorrelated activity. Triangles mark coinciding 1-min bursts of firing rate. D2. Firing rates of neurons during the period of circadian peaks with correlated FOFR.

Reprinted from Neurosciense Research v75, Kononenko, N. I., S. Honma, and K. Honma. Fast synchronous oscillations of firing rate in cultured rat suprachiasmatic nucleus neurons: possible role in circadian synchronization in the intact nucleus. Pp 218-227. Copyright (2013), with permission from Elsevier.
