# Supplementary material for: A Model for the Fast Synchronous Oscillations of Firing Rate in Rat Suprachiasmatic Nucleus Neurons Cultured in a Multielectrode Array Dish
Source: PLoS One. 2014 Sep 5;9(9):e106152. doi: 10.1371/journal.pone.0106152 (PMC4156468; doi:10.1371/journal.pone.0106152)
Supplement: Figure S2 — Synchronization of the circadian activity in the networks of SCN neurons with a random uniform distribution of initial phases of oscillation. (DOC) [file pone.0106152.s003.doc]

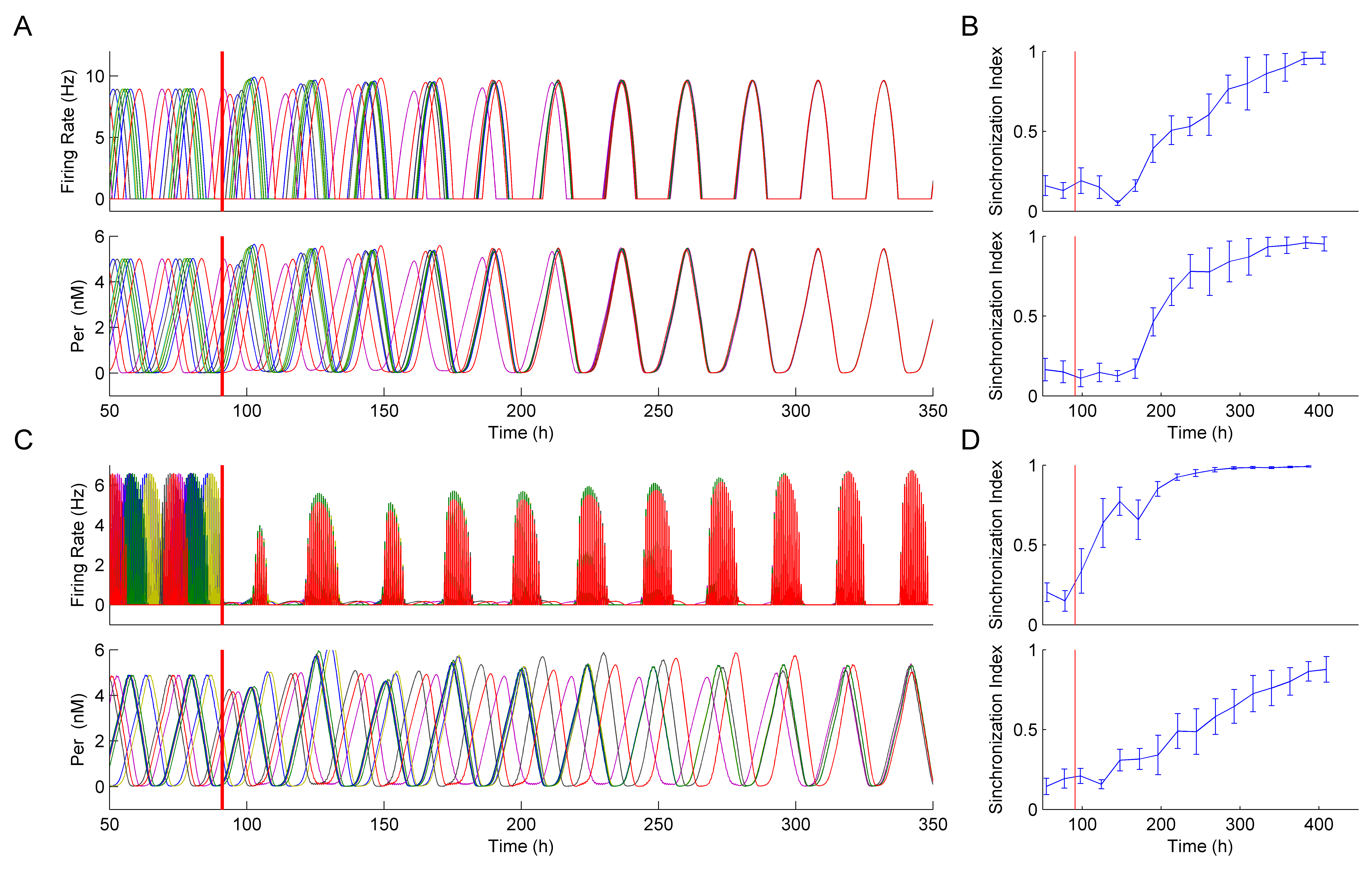


Fig. S2. Synchronization of the circadian activity in the networks of SCN neurons with random uniform distribution of initial phases of oscillation.

A. Firing rates (top) and Per expression (bottom) before and after introduction of VIP exchange (red line) in the network of 10 cells without FOFR (1^st^ model, 1^st^ experiment, see Methods). All cells had default sets of parameters of both circadian clock and fast oscillations models. Circadian discharges started with phase shifts randomly and uniformly distributed within 24h interval and without exchange by VIP (D=0) up to the 91 hour (red line). Then VIP exchange between cells was introduced (D=10). B. The synchronization index averaged over 5 sessions of experiments with different initial distributions of phase.

C,D. The same experiments as in A, B for the 2^nd^ model of interaction of FOFR and circadian oscillations, i.e. when firing rate was regulated mostly by external VIP, through CNG channels co localized with VPAC_2_ receptors, but with influence of Per product on the conductivity of the minor fraction of CNG channels (see Methods).

In the additional series of experiments, putative role of FOFR mechanism in general synchronization of electrical circadian activity in cellular network was investigated in simulations with maximally broad (24-h) random distributions of initial phases of circadian oscillations (Fig. 8). Firstly, in model using regulation of electrical activity exclusively via activation of CNG-channels by *Per* gene product (Fig. 8A), synchronization of circadian electrical firing peaks and *Per* gene product was achieved approximately simultaneously with half-time of about 120 hrs after switching on VIP exchange (Fig. 8B). At the same time, in model using regulation of electrical activity via both activation of CNG-channels by *Per* gene product (20% of total pool of CNG-channels) and activation of CNG-channels through G_s_/AC/cAMP/CNG-channels pathway (80% of total pool of CNG-channels) (Fig. 8C), synchronization of electrical firing was achieved with a half-time of about 25 hrs while half-time of synchronization of *Per* gene product was about 140 hrs (Fig. 8D).
